# Chemical Constituents and Biological Activities of *Bruguiera* Genus and Its Endophytes: A Review

**DOI:** 10.3390/md22040158

**Published:** 2024-03-29

**Authors:** Xiongming Luo, Xiaohong Chen, Lingli Zhang, Bin Liu, Lian Xie, Yan Ma, Min Zhang, Xiaobao Jin

**Affiliations:** 1 School of Life Sciences and Biopharmaceutics, Guangdong Pharmaceutical University, Guangzhou 510006, China; cxh2sky@163.com (X.C.); gdpu_zll@163.com (L.Z.); vert2222@163.com (B.L.); chain1009@163.com (L.X.); 2 Guangdong Provincial Key Laboratory of Pharmaceutical Bioactive Substances, Guangdong Pharmaceutical University, Guangzhou 510006, China; mayan@gdpu.edu.cn (Y.M.); zhangminlala@126.com (M.Z.); 3 School of Basic Medical Sciences, Guangdong Pharmaceutical University, Guangzhou 510006, China

**Keywords:** *Bruguiera*, endophyte, chemical constituents, bioactivities, interaction relationship

## Abstract

The genus *Bruguiera*, a member of the Rhizophoraceae family, is predominantly found in coastal areas as a mangrove plant, boasting a rich and diverse community of endophytes. This review systematically compiled approximately 496 compounds derived from both the *Bruguiera* genus and its associated endophytes, including 152 terpenoids, 17 steroids, 16 sulfides, 44 alkaloids and peptides, 66 quinones, 68 polyketides, 19 flavonoids, 38 phenylpropanoids, 54 aromatic compounds, and 22 other compounds. Among these, 201 compounds exhibited a spectrum of activities, including cytotoxicity, antimicrobial, antioxidant, anti-inflammatory, antiviral, antidiabetic, insecticidal and mosquito repellent, and enzyme inhibitory properties, etc. These findings provided promising lead compounds for drug discovery. Certain similar or identical compounds were found to be simultaneously present in both *Bruguiera* plants and their endophytes, and the phenomenon of their interaction relationship was discussed.

## 1. Introduction

Mangroves thriving in tropical and subtropical coastal regions, estuaries, and mudflats represent unique plant communities. Globally, these mangrove plants host a diverse distribution of 84 species belonging to 24 genera and 16 families [1]. The Rhizophoraceae family, in particular, encompasses approximately four genera, with *Bruguiera* being one of them [2]. Currently, the *Bruguiera* genus comprises six species, including *Bruguiera cylindrica*, *B. exaristata*, *B. gymnorrhiza*, *B. hainessi*, *B. parviflora*, and *B. sexangula*, which are mainly distributed along the coasts of Asia, the islands of the eastern Pacific Ocean, and the coasts of Oceania, and one variety *B. sexangula var. rhynchopetala*, found in the Hainan and Guangdong provinces of China [2,3,4]. It has been reported that *B. gymnorrhiza*, *B. cylindrica*, *B. sexangula*, and *B. parviflora*, have undergone pharmacological validation for their role as traditional medicinal plants [5]. Among these, *B. gymnorrhiza* stands out as the most extensively used for traditional purposes. In diverse regions such as the Sundarbans, the Pichavaram and Pichavaram forests of India, Guangxi province of China, South Andaman Island, Selangor of Malaysia, Indonesia, and the Comoros of Mauritius, inhabitants leverage different components of *B. gymnorrhiza* to address various health conditions [5]. For example, the bark and leaf were employed for treating diarrhea, malaria, fevers, burns, diabetes, liver disorders, and intestinal worms [6,7]. The root was found to have applications in the treatment of diabetes and hyperlipidemias [8]. The fruit is utilized for managing conditions like shingles, eye diseases, and diarrhea [9]. Additionally, the bark of *B. cylindrica* demonstrates efficacy in treating hemorrhages and ulcers [10], while the bark of *B. parviflora* has been utilized in diabetes treatment [5].

In 1966, Loder and Russell first reported the isolation of a new tropane alkaloid brugine from the stem bark of *B. sexangula* [11]. In 2008, Wu et al. conducted the first comprehensive review of natural products from true mangrove flora worldwide, encompassing their sources, chemistries, and bioactivities [3]. In 2014, Zheng et al. compiled 38 chemical constituents of the *Bruguiera* genus up to 2010 [12]. Subsequently, in 2018, Xie et al. conducted a literature search spanning from 1972 to 2017, providing an overview of the chemical compositions and biological activities of *B. gymnorrhiza* [13]; however, certain inaccuracies were identified. In 2021, Chen et al. summarized 1387 secondary metabolites from fungi associated with mangroves, covering the period from 1989 to 2020, detailing their sources, chemistries, and biological activities [14]. Our research team has undertaken partial studies on the isolation and activity screening of secondary metabolites from mangrove plants and marine microorganisms [15,16,17,18,19]. Currently, we are engaged in relevant research on the isolation and activity screening of secondary metabolites from *Bruguiera* plants and their endophytes.

The ongoing isolation endeavors for plants belonging to this genus are predominantly centered around four specific species: *B. gymnorrhiza*, *B. cylindrica*, *B. sexangula*, and *B. sexangula var. rhynchopetala*. A total of 167 secondary metabolites have been unearthed, with a unique class of sulfur-containing compounds discovered particularly in *B. gymnorrhiza*. Starting from 2018, the isolation of novel compounds from plants within the *Bruguiera* genus has encountered escalating challenges, and the enthusiasm for research in this area has gradually diminished. However, there is a current research focus on the secondary metabolites produced by endophytes within the *Bruguiera* genus.

The unique habitat of mangroves has nurtured a rich resource of fungi, bacteria, actinomycetes, and other microorganisms, particularly mangrove associated fungi, which constitute the second largest group of marine fungi [14,20]. These fungi play a crucial role in regulating the mangrove ecosystem, and produce unique structural and diverse bioactive secondary metabolites [14]. Since 2007, researchers have demonstrated considerable interest in the exploration of structurally novel and bioactive compounds derived from endophytes of mangrove genus *Bruguiera*. These endophytes, comprising fungi, bacteria, and actinomycetes, are predominantly sourced from various plant components, including branch, leaf, stem, bark, root, hypocotyl, and inner tissue. To better exploit the medicinal potential of microbial resources, researchers have extensively cultured and isolated endophytic fungi within the *Bruguiera* genus, conducting analyses on both their secondary metabolites and biological activities. Through a compilation of the relevant literature, it was revealed that these endophytes comprise 34 fungi strains, belonging to 19 genera (strains), namely *Aspergillus* (4), *Cladosporium* (1), *Clonostachys* (1), *Daldinia* (2), *Epicoccum* (1), *Fusarium* (1), *Gloesporium* (1), *Nigrospora* (1), *Nectria* (1), *Peniophora* (1), *Penicillium* (10), *Pestalotiopsis* (2), *Pseudolagarobcasidium* (1), *Phomopsis* (1), *Phyllosticta* (1), *Rhytidhysteron* (1), *Stemphylium* (1), *Trichoderma* (1), and *Xylaria* (2), along with four actinomycetes, affiliated with the genus *Streptomyces* (4). The above 38 strains of endophytes were derived from *B. gymnorrhiza* (63.2%), *B. sexangula var. rhynchopetala* (21.0%), *B. sexangula* (13.2%), and *B. parviflora* (2.6%). 

Currently, 337 secondary metabolites have been found from the endophytes of the *Bruguiera* genus. Some of these compounds exhibit rare structural features and demonstrate significant bioactivity. For example, in the study by Zhang et al. [21], four novel 12-membered macrocyclic lactone compounds (**179–182**) with sulfur substitution at C-2 were obtained from the endophytic fungus *Cladosporium cladosporioides* MA-299, isolated from the leaves of *B. gymnorrhiza*, and the compounds exhibited notable antimicrobial activity against aquatic bacteria (*Edwardsiella tarda* and *Edwardsiella ictarda*) and plant pathogens (*Bipolaris sorokiniana*, *Colletotrichum glecosporioides*, *Fusarium oxysporum* f. sp. *cucumerinum*, and *Physalospora piricola* Nose). Additionally, Xu et al. [22] got a range of structurally diverse ansamycin compounds, named divergolides A–D (**329–332**), from the endophytic actinomycete *Streptomyces sp.* in *B. gymnorrhiza*, offering a detailed analysis of the polyketide synthase domain, which not only validated the stereochemical integrity of divergolides, but also yielded valuable insights into the formation mechanisms of the diverse aromatic chromophores. Evidently, endophytes of *Bruguiera* genus plants emerge as a promising reservoir for acquiring structurally novel compounds.

Research indicates that there is no significant distinction in marine fungal natural products obtained from different ecological niches [23]. In-depth exploration of understudied microbial phyla, particularly those unique to specific environments, is more likely to unveil structurally novel biologically active compounds [24]. Clearly, there is still a substantial knowledge gap in the current exploratory research on endophytic bacteria and actinomycetes within the *Bruguiera* genus, necessitating further in-depth investigation. Simultaneously, a significant amount of current research employs similar isolation and cultivation techniques targeting microorganisms of the same genus, leading to the production of structurally similar or the known compounds during the processes of microbial culture and secondary metabolite isolation [25]. Therefore, to extract a more diverse array of unique natural products from endophytes within the *Bruguiera* genus, we should adopt a targeted approach. This involves the targeted isolation of strains, guided by early genomic sequencing to inform strain selection, and innovations in culture media and cultivation techniques [25].

This review encompasses the relevant literature describing the chemical composition and biological activities of *Bruguiera* genus plants and their endophytes until 30 December 2023. It summarizes approximately 496 compounds, of which 201 exhibit biological activity. Of particular note, during the process of the literature organization, it was observed that certain compounds—such as cytochalasin D, zygosporin D, 2,6-dimethoxy-1,4-benzoquinone, scopoletin, and so on—either coexist in both plants and their endophytic fungi or play a crucial regulatory role in the interaction between plants and endophytic fungi. The noteworthy discovery will be further explored and discussed below.

## 2. Chemical Composition of *Bruguiera* Genus Plants and Their Endophytes

**Figure 1:**
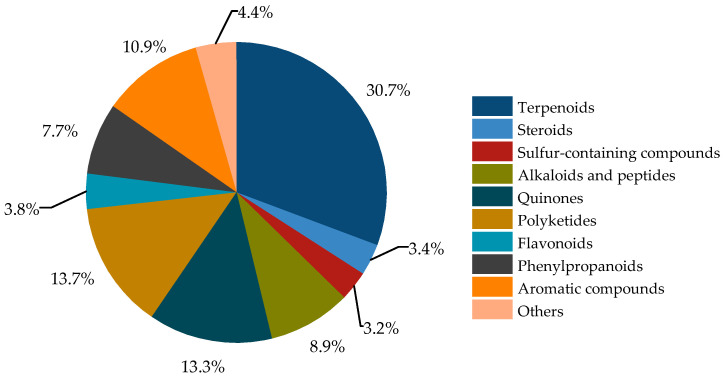
Classification of secondary metabolites from plants of *Bruguiera* genus and their endophytes.

The secondary metabolites of *Bruguiera* genus plants and their endophytes demonstrate a diverse array (Figure 1), comprising terpenoids (30.7%), steroids (3.4%), sulfur-containing compounds (3.2%), alkaloids and peptides (8.9%), quinones (13.3%), polyketides (13.7%), flavonoids (3.8%), phenylpropanoids (7.7%), aromatic compounds (10.9%), and other compounds (4.4%).

### 2.1. Terpenoids

Terpenoids constitute the largest category of chemical constituents in both *Bruguiera* genus plants and their endophytes, totaling 152 compounds. This diverse group includes 2 monoterpenes, 56 sesquiterpenes, 28 diterpenes, 4 sesterterpenes, 40 triterpenes, and 22 meroterpenes. Monoterpenes, sesterterpenes, and meroterpenes are exclusively found in endophytic fungi. Diterpenes are isolated only from *Bruguiera* plants, which suggests the potential absence or inhibition of the biosynthetic pathway for these compounds in their endophytes.

#### 2.1.1. Monoterpenes

**Figure 2:**
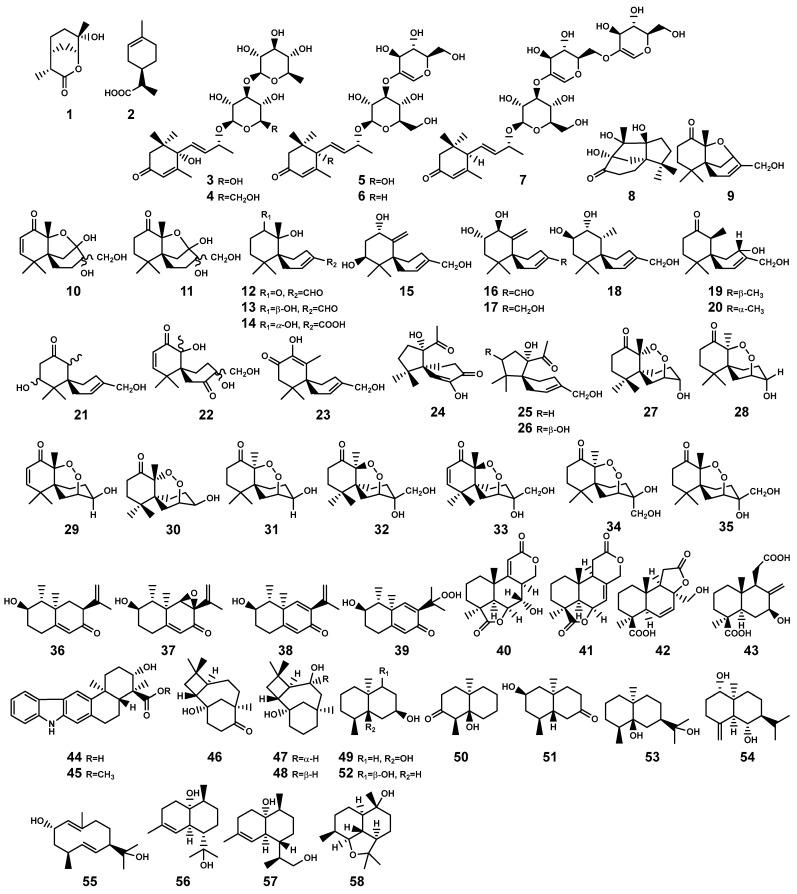
Monoterpenes (**1**–**2**) and sesquiterpenes (**3**–**58**) isolated from *Bruguiera* genus plants and their endophytes.

**Figure 3:**
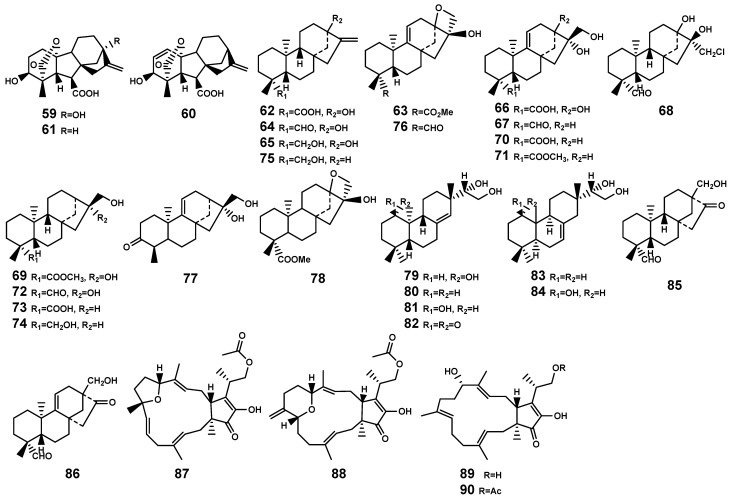
Diterpenes (**59**–**86**) and sesterterpenes (**87**–**90**) isolated from *Bruguiera* genus plants and their endophytes.

**Figure 4:**
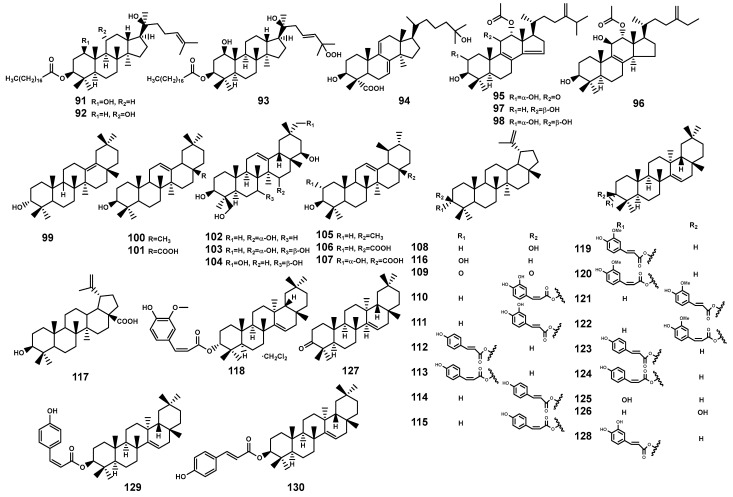
Triterpenoids (**91**–**130**) isolated from *Bruguiera* genus plants and their endophytes.

In the context of *Bruguiera* genus plants and their endophytes, the occurrence of monoterpenoid compound is infrequent. Only Xu et al. [26] isolated two antibacterially active monoterpenoid derivatives (Figure 2), (3*R*,4*R*,6*R*,7*S*)-7-hydroxyl-3,7-dimethyl-oxabicyclo[3.3.1]nonan-2-one (**1**) and (3*R*,4*R*)-3-(7-methylcyclohexenyl)-propanoic acid (**2**), from the fermentation product of *Pestalotiopsis foedan*, an endophytic fungus found in the branch of *B. sexangula*. While a variety of monoterpenes, such as trans-*β*-ocimene and linalool, are present in the essential oils of *Bruguiera* plants like the flower of *B. gymnorrhiza* [27], researchers have seldom conducted isolation studies on this particular fraction of crude extracts.

#### 2.1.2. Sesquiterpenes

In the *Bruguiera* genus and their endophytes, the biosynthetic pathway for sesquiterpenoids is exclusively present in both *B. gymnorrhiza* plant and its endophytes, having the production of 56 sesquiterpenoid compounds (**3–58**, Table 1 and Figure 2). In a study by Wibowo et al. [28,29], a total of 28 (nor)sesquiterpens (**8–35**) were isolated from the endophytic fungus *Pseudolagarobasidium acaciicola* in the roots of *B. gymnorrhiza*. Compound **8** is a novel tricyclic norsesquiterpene with a unique acaciicolane skeleton featuring a 6/5/5 ring system. Compounds **24–26** are identified as new spirobicyclic (nor)sesquiterpenes, characterized by a spiroacaciicolane skeleton with a 5/6 fused spirobicyclic ring system [28,29]. (Nor)sesquiterpenoid compounds (**27–29**, **35**) are found as new nor-chamigrane endoperoxides [28,29]. Additionally, compounds **9–11** and **12–23** possess a novel 6/5/6 tricyclic ring system and a 6/6 spirobicyclic structure, respectively [29]. 

Ding and colleagues [30,31,32,33] identified five types of sesquiterpenes from two endophytic actinomycetes, *Streptomyces sp.* GT2002/1503 and *Streptomyces sp.* JMRC:ST027706, isolated from the stems of *B. gymnorrhiza*. These compounds encompass indolosesquiterpenes (**44–45**), plant-derived caryolanes (**46–48**), geosmins (**49–52**), plant-like eudesmanes (**53–55**), and plant-like cadinanes (**56–58**). Significantly, among these, compound **53** demonstrated noteworthy broad antimicrobial activity and also emerged as a principal constituent in the essential oil derived from the aromatic grass *Cymbopogon distans*, which may play a pivotal role as an active ingredient in the medicinal plant *C. distans* [32]. Nevertheless, there exists a plausible suspicion that this compound may also be produced by *Streptomyces sp.* JMRC:ST027706 in a manner to the plant’s defense response when the host *B. gymnorrhiza* plant is subjected to pathogenic threats. This implies a potential collaborative mechanism where the endophytic actinomycete aids the host plant in bolstering its resistance against diseases. Moreover, the discovery of oxygenated geosmins is interesting, as it has brought to light the potential presence of enzymes within the endophytic actinomycetes capable of modifying geosmin oxidizing the decalin core structure found in geosmin and analogous terpenoids [32]. This revelation not only expands our understanding but also introduces a promising prospect for employing biotechnological strategies to harness these enzymes for geosmin degradation.

**Table 1:** Sesquiterpenes isolated from *Bruguiera* genus plants and their endophytes.

No.	Compound	Source	Reference
3	(*S*)-4-hydroxy-3,5,5-trimethyl-4-((*R*,*E*)-3-(((2*R*,3*R*,4*S*,5*S*,6*S*)-3,5,6-trihydroxy-4-(((2*S*,3*R*,4*S*,5*S*,6*R*)-3,4,5-trihydroxy-6methyltetrahydro-2*H*-pyran-2-yl)oxy)tetrahydro-2*H*-pyran-2-yl)oxy)but-1-en-1-yl)cyclohex-2-en-1-one	*B. gymnorrhiza*, leaf	[34]
4	(*S*)-4-((*R*,*E*)-3-(((2*R*,3*R*,4*S*,5*R*,6*R*)-3,5-dihydroxy-6-(hydroxymethyl)-4-(((2*S*,3*R*,4*S*,5*S*,6*R*)-3,4,5-trihydroxy-6-methyltetrahydro-2*H*-pyran-2-yl)oxy)tetrahydro-2H-pyran-2-yl)oxy)but-1-en-1-yl)-4-hydroxy-3,5,5-trimethylcyclohex-2-en-1-one	*B. gymnorrhiza*, leaf	[34]
5	(*S*)-4-((*R*,*E*)-3-(((2*R*,3*R*,4*S*,5*R*,6*R*)-4-(((2*R*,3*S*,4*S*)-3,4-dihydroxy-2-(hydroxymethyl)-3,4-dihydro-2*H*-pyran-5-yl)oxy)-3,5-dihydroxy-6-(hydroxymethyl)tetrahydro-2*H*-pyran-2-yl)oxy)but-1-en-1-yl)-4-hydroxy-3,5,5-trimethylcyclohex-2-en-1-one	*B. gymnorrhiza*, leaf	[34]
6	(*R*)-4-((*R*,*E*)-3-(((2*R*,3*R*,4*S*,5*R*,6*R*)-4-(((2*R*,3*S*,4*S*)-3,4-dihydroxy-2-(hydroxymethyl)-3,4-dihydro-2*H*-pyran-5-yl)oxy)-3,5-dihydroxy-6-(hydroxymethyl)tetrahydro-2*H*-pyran-2-yl)oxy)but-1-en-1-yl)-3,5,5-trimethylcyclohex-2-en-1-one	*B. gymnorrhiza*, leaf	[34]
7	(*R*)-4-((*R*,*E*)-3-(((2*R*,3*R*,4*S*,5*R*,6*R*)-4-(((2*R*,3*S*,4*S*)-2-((((2*R*,3*S*,4*S*)-3,4-dihydroxy-2-(hydroxymethyl)-3,4-dihydro-2*H*-pyran-5-yl)oxy)methyl)-3,4-dihydroxy-3,4-dihydro-2H-pyran-5-yl)oxy)-3,5-dihydroxy-6-(hydroxymethyl)tetrahydro-2*H*-pyran-2-yl)oxy)but-1-en-1-yl)-3,5,5-trimethylcyclohex-2-en-1-one	*B. gymnorrhiza*, leaf	[34]
8	Acaciicolin A	*P. acaciicola* (the root of *B. gymnorrhiza*, endophytic fungus)	[28]
9	Acaciicolide A	*P. acaciicola* (the root of *B. gymnorrhiza*, endophytic fungus)	[29]
10	Acaciicolide B	*P. acaciicola* (the root of *B. gymnorrhiza*, endophytic fungus)	[29]
11	Acaciicolide C	*P. acaciicola* (the root of *B. gymnorrhiza*, endophytic fungus)	[29]
12	Acaciicolinol A	*P. acaciicola* (the root of *B. gymnorrhiza*, endophytic fungus)	[29]
13	Acaciicolinol B	*P. acaciicola* (the root of *B. gymnorrhiza*, endophytic fungus)	[29]
14	Acaciicolinol C	*P. acaciicola* (the root of *B. gymnorrhiza*, endophytic fungus)	[29]
15	Acaciicolinol D	*P. acaciicola* (the root of *B. gymnorrhiza*, endophytic fungus)	[29]
16	Acaciicolinol E	*P. acaciicola* (the root of *B. gymnorrhiza*, endophytic fungus)	[29]
17	Acaciicolinol F	*P. acaciicola* (the root of *B. gymnorrhiza*, endophytic fungus)	[29]
18	Acaciicolinol G	*P. acaciicola* (the root of *B. gymnorrhiza*, endophytic fungus)	[29]
19	Acaciicolinol H	*P. acaciicola* (the root of *B. gymnorrhiza*, endophytic fungus)	[29]
20	Acaciicolinol I	*P. acaciicola* (the root of *B. gymnorrhiza*, endophytic fungus)	[29]
21	Acaciicolinol J	*P. acaciicola* (the root of *B. gymnorrhiza*, endophytic fungus)	[29]
22	Acaciicolinol K	*P. acaciicola* (the root of *B. gymnorrhiza*, endophytic fungus)	[29]
23	Acaciicolinol L	*P. acaciicola* (the root of *B. gymnorrhiza*, endophytic fungus)	[29]
24	Spiroacaciicolide A	*P. acaciicola* (the root of *B. gymnorrhiza*, endophytic fungus)	[28]
25	Spiroacaciicolide B	*P. acaciicola* (the root of *B. gymnorrhiza*, endophytic fungus)	[29]
26	Spiroacaciicolide C	*P. acaciicola* (the root of *B. gymnorrhiza*, endophytic fungus)	[29]
27	3-epi-Steperoxide A	*P. acaciicola* (the root of *B. gymnorrhiza*, endophytic fungus)	[28]
28	3-epi-merulin A	*P. acaciicola* (the root of *B. gymnorrhiza*, endophytic fungus)	[29]
29	(3*S*,4*R*,6a*S*,10a*R*)-4-hydroxy-7,7,10a-trimethyl-5,6,7,10a-tetrahydro-3*H*-3,6a-methanobenzo[c] [1,2] dioxocin-10(4*H*)-one	*P. acaciicola* (the root of *B. gymnorrhiza*, endophytic fungus)	[29]
30	Steperoxide A	*P. acaciicola* (the root of *B. gymnorrhiza*, endophytic fungus)	[28]
31	Merulin A	*P. acaciicola* (the root of *B. gymnorrhiza*, endophytic fungus)	[29]
32	Merulin B	*P. acaciicola* (the root of *B. gymnorrhiza*, endophytic fungus)	[28]
33	Merulin C	*P. acaciicola* (the root of *B. gymnorrhiza*, endophytic fungus)	[28]
34	Merulin D	*P. acaciicola* (the root of *B. gymnorrhiza*, endophytic fungus)	[29]
35	7-epi-merulin B	*P. acaciicola* (the root of *B. gymnorrhiza*, endophytic fungus)	[29]
36	Petasol	*B. gymnorrhiza*	[35]
37	Sporogen AO1	*B. gymnorrhiza*	[35]
38	6-dehydropetasol	*B. gymnorrhiza*	[35]
39	3*α*-hydroxy-11-peroxyl-eremophila-6,9-dien-8-one	*B. gymnorrhiza*	[35]
40	Botryosphaerin F	*Aspergillus terreus* No. GX7-3B (the branch of *B. gymnorrhiza*, endophytic fungus)	[36]
41	13,14,15,16-tetranorlabd-7-ene-19,6b:12,17-diolide	*A. terreus* No. GX7-3B (the branch of *B. gymnorrhiza*, endophytic fungus)	[36]
42	Botryosphaerin B	*A. terreus* No. GX7-3B (the branch of *B. gymnorrhiza*, endophytic fungus)	[36]
43	LLZ1271*β*	*A. terreus* No. GX7-3B (the branch of *B. gymnorrhiza*, endophytic fungus)	[36]
44	Xiamycin	*Streptomyces sp.* GT2002/1503 (the stem of *B. gymnorrhiza*, endophytic actinomycete)	[30]
45	Methyl ester of xiamycin	*Streptomyces sp.* GT2002/1503 (the stem of *B. gymnorrhiza*, endophytic actinomycete)	[30]
46	Bacaryolane A	*Streptomyces sp.* JMRC:ST027706 (the stem of *B. gymnorrhiza*, endophytic actinomycete)	[31]
47	Bacaryolane B	*Streptomyces sp.* JMRC:ST027706 (the stem of *B. gymnorrhiza*, endophytic actinomycete)	[31]
48	Bacaryolane C	*Streptomyces sp.* JMRC:ST027706 (the stem of *B. gymnorrhiza*, endophytic actinomycete)	[31]
49	7*R*-hydroxygeosmin	*Streptomyces sp.* JMRC:ST027706 (the stem of *B. gymnorrhiza*, endophytic actinomycete)	[32]
50	3-oxogeosmin	*Streptomyces sp.* JMRC:ST027706 (the stem of *B. gymnorrhiza*, endophytic actinomycete)	[32]
51	2*R*-hydroxy-7-oxogeosmin	*Streptomyces sp.* JMRC:ST027706 (the stem of *B. gymnorrhiza*, endophytic actinomycete)	[32]
52	5-deoxy-7*β*,9*β*-dihydroxygeosmin	*Streptomyces sp.* JMRC:ST027706 (the stem of *B. gymnorrhiza*, endophytic actinomycete)	[32]
53	(4*S*,5*S*,7*R*,10*S*)-4*β*,10*α*-eudesmane-5*β*,11-diol	*Streptomyces sp.* JMRC:ST027706 (the stem of *B. gymnorrhiza*, endophytic actinomycete)	[32]
54	(1*S*,5*S*,6*S*,7*S*,10*S*)-10*α*-eudesm-4(15)-ene-1*α*,6*α*-diol	*Streptomyces sp.* JMRC:ST027706 (the stem of *B. gymnorrhiza*, endophytic actinomycete)	[32]
55	1(10)*E*,5*E*-germacradiene-2,11-diol	*Streptomyces sp.* JMRC:ST027706 (the stem of *B. gymnorrhiza*, endophytic actinomycete)	[32]
56	(+)-11-hydroxy-epicubenol	*Streptomyces sp.* JMRC:ST027706 (the stem of *B. gymnorrhiza*, endophytic actinomycete)	[33]
57	(+)-12-hydroxy-epicubenol	*Streptomyces sp.* JMRC:ST027706 (the stem of *B. gymnorrhiza*, endophytic actinomycete)	[33]
58	5,11-epoxy-10-cadinanol	*Streptomyces sp.* JMRC:ST027706 (the stem of *B. gymnorrhiza*, endophytic actinomycete)	[33]

#### 2.1.3. Diterpenoids

Diterpenoids are a class of compounds derived from the precursor (geranylgeranyl diphosphate, GGPP) [37]. The diversity of diterpenoids in plants playing important roles in plant development, stress resistance, and interactions with environmental microorganisms, depends on the various skeletons biosynthesized by terpene synthases and the substrate promiscuity of cytochrome P450 monooxygenases (P450s), along with other post-modification enzymes [38]. In the genus *Bruguiera*, researchers have isolated five different skeletal types of diterpenoid (Table 2 and Figure 3), namely *ent*-pimarane (**79**–**82**), isopimarane (**83**–**84**), *ent*-beyerane (**85**–**86**), *ent*-kaurane (**62**–**78**), and *ent*-gibberellane (**59**–**61**), with the key intermediate pimarane in their biosynthesis.

#### 2.1.4. Sesterpenoids

Most sesterpenoids are sourced from marine organisms, with approximately 15% of these compounds having been isolated from plants [44]. Currently, no such compounds have been identified in *Bruguiera* plants, emphasizing the need for further exploration in this area. Only Liu et al. [45] reported the discovery of two tricyclic sesterterpenes, fusaprolifin A and fusaprolifin B (**87–88**) with brine-shrimp lethality activity, and other two sesterterpenes, terpestacin and fusaproliferin (**89–90**), which isolated from the endophytic fungus *Fusarium proliferatum* MA-84 associated with *B. sexangula* plant (Figure 3).

#### 2.1.5. Triterpenoids

In the *Bruguiera* plants and their endophytic fungi, triterpenoids (**91**–**130**, Table 3 and Figure 4) constitute a primary class of compounds. Distinguished by variations in carbon ring structures, there are tetracyclic triterpenoids, such as dammarane-type (**91**–**93**) and lanostane-type (**94–98**). Additionally, pentacyclic triterpenoids are present, including oleanane-type (**99**–**104**), ursane-type (**105**–**107**), lupane-type (**108–117**), and other types (**118–130**). 

#### 2.1.6. Meroterpenoids

**Figure 5:**
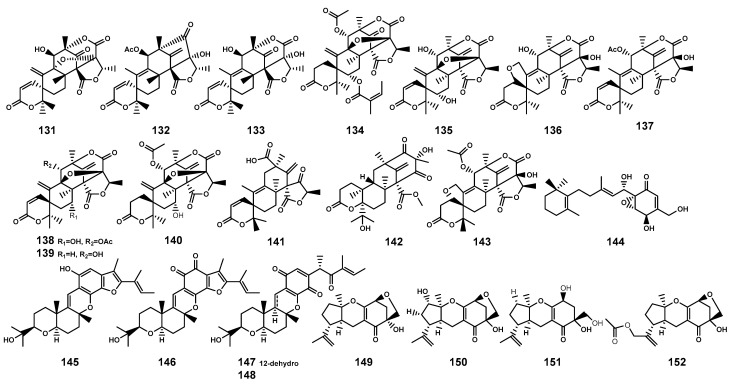
Meroterpenoids (**131**–**152**) isolated from *Bruguiera* genus plants and their endophytes.

Meroterpenoids as secondary metabolites arising from hybrid terpenoid biosynthetic pathways, are characterized by the combination of terpenoid and non-terpenoid segments [59]. Based on their non-terpenoid starting moieties, these compounds were categorized into four classes: polyketide–terpenoids, indole–terpenoids, shikimate–terpenoids, and miscellaneous meroterpenoids [59]. This review summarized 22 meroterpenoids (Table 4 and Figure 5) isolated from the endophytic fungi found in *B. gymnorrhiza*, *B. sexangula*, and *B. sexangula var. rhynchopetala*. These meroterpenoids have been classified as shikimate-meroterpenoids of the tricycloalternarene type (**149**–**152**) and polyketide-meroterpenoids (**131**–**148**), which contain tetraketide moieties derived from 3,5-dimethylorsellinic acid (**131**–**143**) and 6-methylsalicylic acid (**144**), and hexaketide moiety (**145**–**148**), respectively. 

### 2.2. Steroids

**Figure 6:**
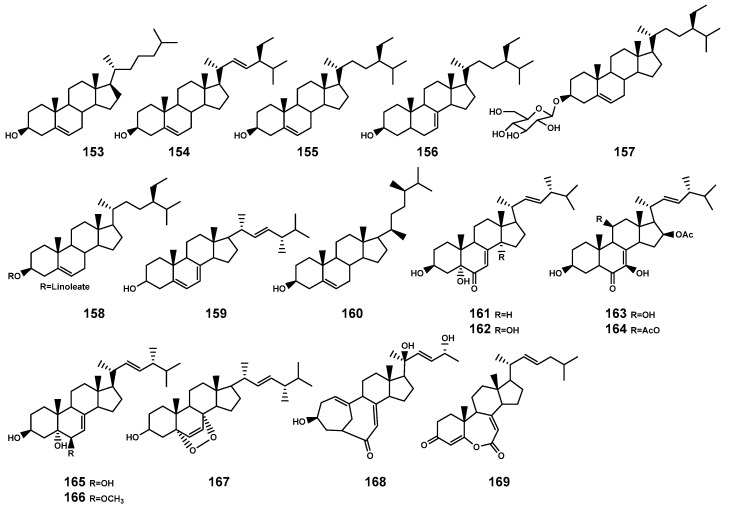
Steroids (**153**–**169**) isolated from *Bruguiera* genus plants and their endophytes.

In plants and fungi, squalene serves as a precursor that undergoes oxidation catalyzed by squalene epoxidase to generate ox1idosqualene [65]. The further cyclization of oxidized squalene, catalyzed by oxidosqualene cyclases, leads to the biosynthesis of sterols representing a significant downstream structural derivative in the biosynthesis of triterpenoids [65]. Currently, 17 steroids (Table 5 and Figure 6) have been identified in the *Bruguiera* plants and their endophytic fungi. These compounds encompass three types: cholestrol (**153**), stigmastanol (**154–158**), and ergosterol (**159–169**), specifically noting the significance of two ergostanes (**168–169**) with a rearranged tetracyclic skeleton.

### 2.3. Sulfides

**Figure 7:**
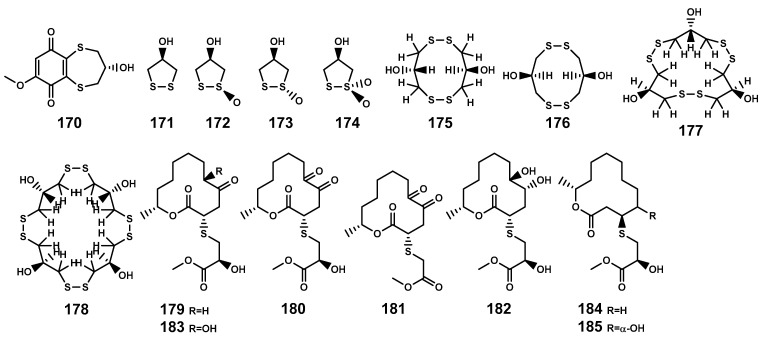
Sulfides (**170**–**185**) isolated from *Bruguiera* genus plants and their endophytes.

**Figure 8:**
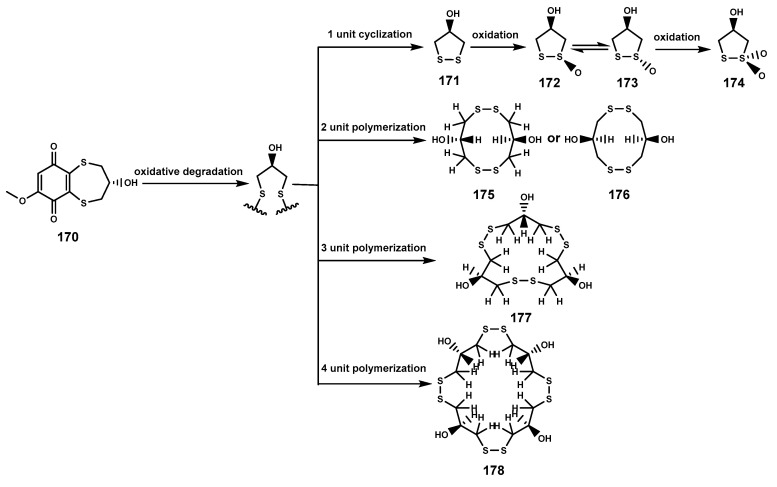
The proposed biosynthetic pathway of disulfide compounds (**170**–**178**).

**Figure 9:**
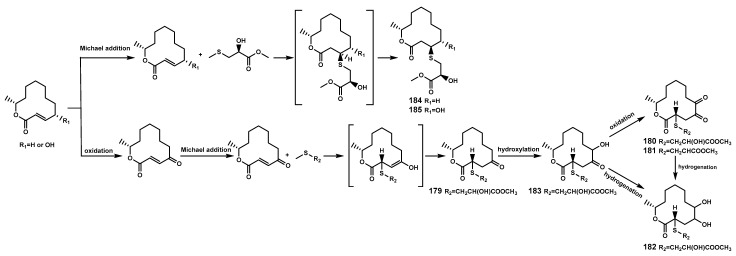
The proposed biosynthetic pathway of sulfur-containing 12-membered macrocyclic lactones (**179**–**185**).

Sulfur-containing natural products are predominantly isolated from plants of the Alliaceae family or bacteria, with relatively fewer sulfur compounds being separated from fungi [71]. Now, this paper summarizes a total of 16 sulfur-containing compounds (Table 6 and Figure 7) from the *Bruguiera* genus of Rhizophoraceae family and their endophytes. Within the plants *B. gymnorrhiza*, *B. sexangula var. rhynchopetala*, and *B. cylindrica*, a class of (poly)disulfide compounds (**170–178**) has been found. These compounds belong to a characteristic type of compounds found in mangrove ecosystem of the *Bruguiera* genus, and exhibit a proposed unique biosynthetic pathway (Figure 8) [41,72]. Previous research indicates that sulfides play a crucial role in plant defense against various pathogens and pests [73]. Dahibhate et al. [74] discovered that a mixture of brugierol and isobrugierol exhibited inhibitory activity against *Pseudomonas aeruginosa* by reducing the formation of virulence biofilms controlled by the quorum sensing system, lowering the level of virulence factors, thereby attenuating the pathogenicity of the bacterium against *Bruguiera* plants. Simultaneously, Zhang et al. [21,75] cultured and isolated the endophytic fungus *C. cladosporioides* MA-299 from the leaves of *B. gymnorrhiza*, obtaining seven sulfur-containing 12-membered macrocyclic lactones (**179–185**). The potential biosynthetic pathway of these compounds is illustrated in Figure 9, with the precursor cladocladosin A featuring the bicyclo 5/9 ring system. These compounds (**179–185**) demonstrate activity against aquatic pathogens and plant-pathogenic fungi [21,75], thereby assisting *B. gymnorrhiza* leaves in resisting microbial pathogen invasion. 

### 2.4. Alkaloids and Peptides

**Figure 10:**
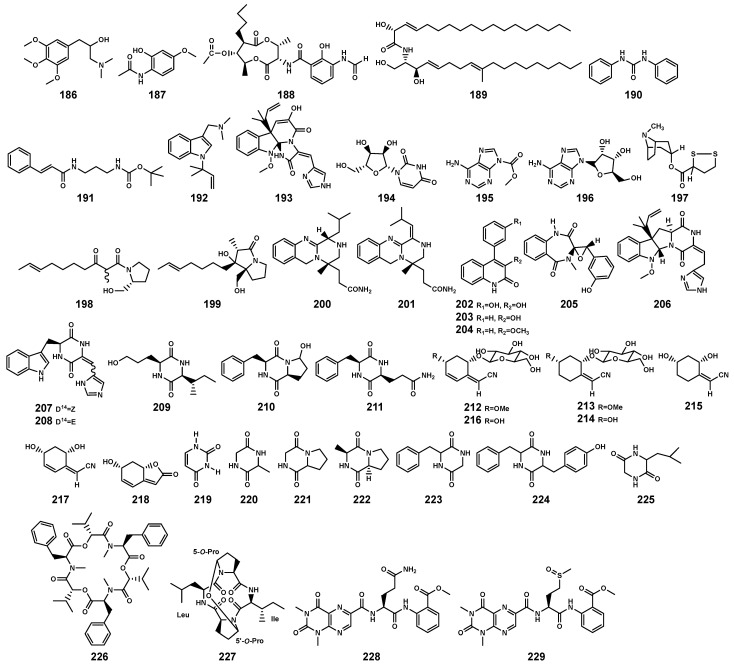
Alkaloids (**186**–**218**) and peptides (**219**–**229**) isolated from *Bruguiera* genus plants and their endophytes.

Alkaloids constitute a class of naturally occurring organic compounds with at least one nitrogen atom, primarily derived from amino acids. They typically exhibit complex cyclic structures, with some playing crucial roles in plant defense mechanisms [81]. Currently, researchers have identified 33 alkaloids (Table 7 and Figure 10) with different types from *B. gymnorrhiza* plant and endophytic fungi in *B. gymnorrhiza* and *B. sexangula var. rhynchopetala*, including amine (**186**) and amide types (**187**–**191**), indole (**192–193**), pyrimidine (**194**) and purine types (**195–196**), tropane (**197**), pyrrolidine (**198**), pyrrolizidine (**199**), quinazoline (**200**–**201**), quinoline (**202–204**), benzodiazepine (**205**), diketopiperazine (**206–211**), and cyclohexylideneacetonitrile derivatives (**212**–**218**). Studies indicate a significant correlation between endophytes and alkaloid [82]. Among the alkaloid-producing endophytic fungi, 66% belong to the *Penicillium* genus, and 75% of the alkaloids isolated from endophytic fungi are derived from the *Penicillium* genus. Clearly, within the endophytic communities of *Bruguiera*, the *Penicillium* genus likely serves as the dominant producer of alkaloids. Additionally, Li et al. [68] isolated a 7-membered 2,5-dioxopiperazine alkaloid (+)-cyclopenol (**205**), from an endophytic fungus *Penicillium sclerotiorum* in the inner bark of *B. gymnorrhiza*. In the terrestrial plant *Garcinia atroviridis*, the endophytic fungus *P. sclerotiorum* produces azaphilone-type derivatives [83]. (-)-cyclopenol, which shares a biosynthetic pathway similar to **205**, was also isolated from terrestrial soil *Penicillium* genus [68,84]. This suggests that the mechanism of (+)-cyclopenol production may be associated with the unique habitat of mangroves.

Compounds **219**–**229** (Table 7 and Figure 10) are peptides derived from endophytic fungi found in *B. gymnorrhiza* and *B. sexangula var. rhynchopetala*, including cyclic dipeptides (**219**–**225**), depsipeptide (**226**), cyclic tetrapeptide (**227**), and lumazines (**228**–**229**).

### 2.5. Quinones

**Figure 11:**
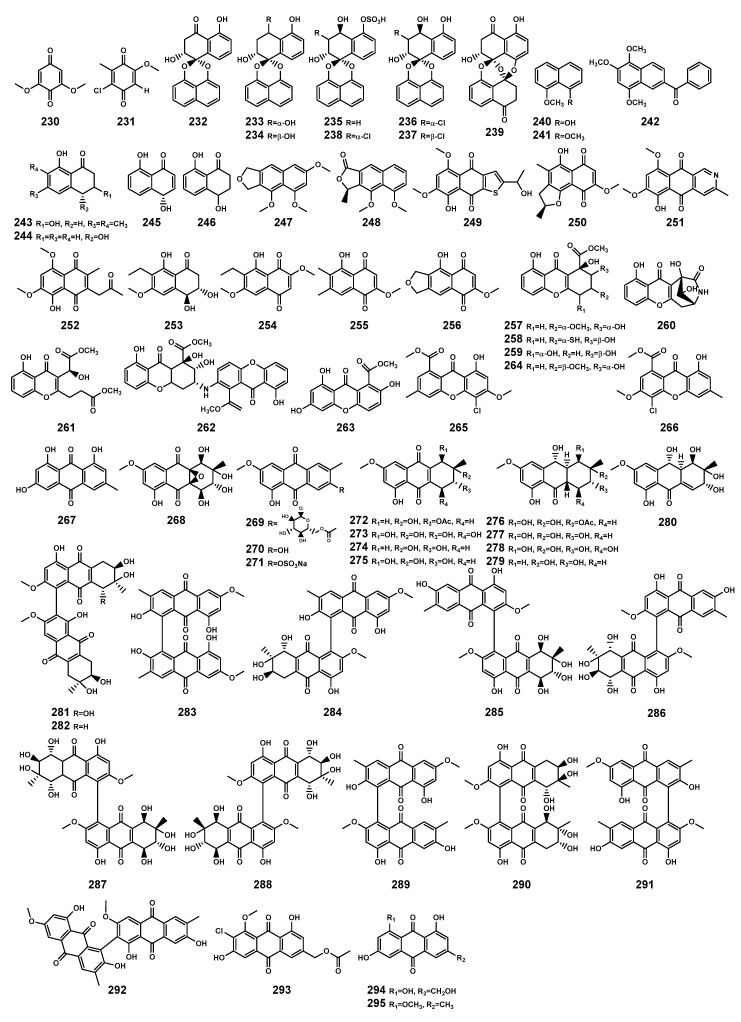
Quinones (**230**–**295**) isolated from *Bruguiera* genus plants and their endophytes.

Plants are the primary producers of quinones [95], but in *Bruguiera* genus plants and their endophytes, 87.5% of quinones originate from endophytic fungi. The identified quinones (Table 8 and Figure 11) include benzoquinones (**230–231**), naphthalenes (**232–242**), naphthalenones (**243**–**246**), naphthofurans (**247**–**248**), naphthoquinones (**249**–**256**), xanthones (**257**–**266**), and anthraquinones (**267**–**295**). Among these, 41.2% of quinones sourced from endophytic fungi exhibit antibacterial activity. Studies have shown that fungi can commonly reduce 2,6-dimethoxy-1,4-benzoquinone (**230**) to hydroquinones, and some fungi showed effects of quinone-dependent polymer polystyrene sulfonate degradation, contributing to driving fungi to utilize the redox cycling of quinones for biodegradation in the ecological environment [96]. In plant immunity, quinone-related molecules could play a role as pathogen- or danger-associated molecular patterns [95]. The series of evidence underscores the ecological significance of quinone substances.

### 2.6. Polyketides

**Figure 12:**
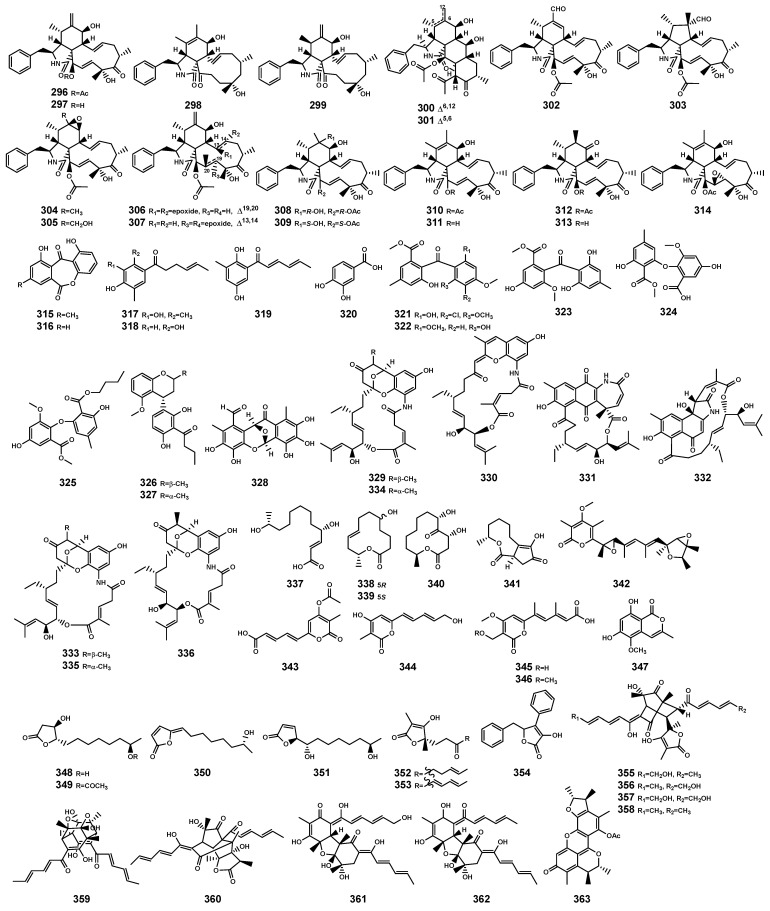
Polyketides (**296**–**363**) isolated from *Bruguiera* genus plants and their endophytes.

In the genus *Bruguiera* and its endophytes, polyketides primarily exhibit antibacterial and cytotoxic activities, predominantly sourced from endophytes of the *Bruguiera* genus. These polyketides (Table 9 and Figure 12) encompass various types, including cytochalasins (**296**–**314**), phenols (**315**–**328**), ansamycins (**329**–**336**), 12-membered macrolides (**337**–**341**), *α*-pyrone (**342–346**), chromanone (**347**), furanones (**348–354**), sorbicillinoids (**355**–**362**), and benzofuranones (**363**). Supratman et al. [108] isolated (-)-dihydrovertinolide (**352**) from endophytic fungus *C. rosea* B5-2 obtained from the branch of *B. gymnorrhiza*. The compound **352** exhibited no antibacterial activity but demonstrated phytotoxicity to lettuce seedlings (*Lactuca sativa* L.) [108]. This effect may enhance the competitive abilities of *B. gymnorrhiza* with other plants in terms of nutrients and space [109], contributing to the reinforcement of *B. gymnorrhiza* plant defense mechanisms. It could serve as a lead compound for the development of potential bioherbicides.

### 2.7. Flavonoids

**Figure 13:**
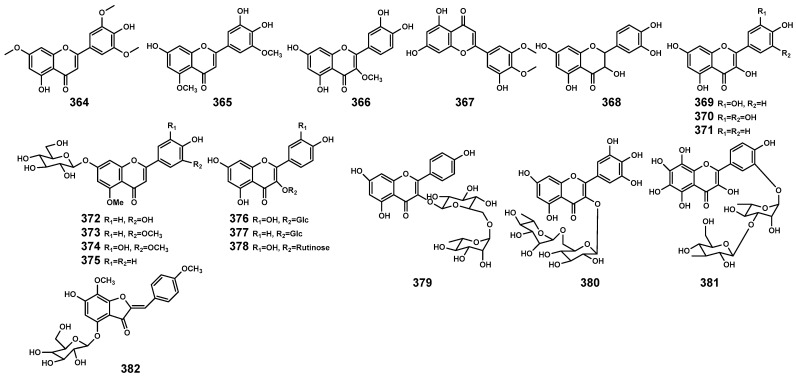
Flavonoids (**364–382**) isolated from *Bruguiera* genus plants and their endophytes.

Flavonoids are commonly present in the organs of plants, participating in the plant’s response to environmental stressors [116]. Currently, in the genus *Bruguiera* and its endophytes, flavonoid (Table 10 and Figure 13) primarily originates from *B. gymnorrhiza* plants, including flavones (**364–367**), flavone glycosides (**372–375**), and flavonol glycosides (**376**–**381**). In the *B. parviflora* plant, dihydroflavonol (**368**) and flavonol (**369–371**) have been identified. Additionally, WU et al. [117] isolated a novel aurone glycoside compound (*Z*)-7,4’-dimethoxy-6-hydroxy-aurone-4-O-*β*-glucopyranoside (**382**) from the endophytic fungus *Penicillium citrinum* associated with *B. gymnorrhiza*, exhibiting significant neuroprotective activity.

### 2.8. Phenylpropanoids

**Figure 14:**
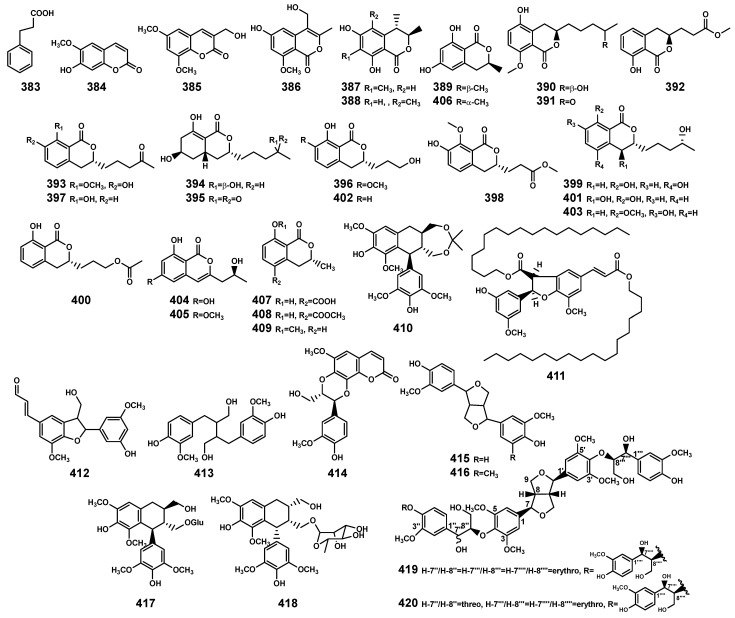
Phenylpropanoids (**383**–**420**) isolated from *Bruguiera* genus plants and their endophytes.

The 38 phenylpropanoids (Table 11 and Figure 14) include phenylpropanoic acid (**383**), coumarins (**384–385**) and isocoumarins (**386**–**409**), and lignans (**410**–**420**). Among these, isocoumarins and lignans are the main compounds, originating from endophytic fungi associated with the *Bruguiera* genus and *Bruguiera* plants, respectively. Previous research has indicated that plant-derived coumarins have the potential to resist infections in both plants and animals [122], particularly scopoletin (**384**). When different plant species are exposed to multiple pathogens such as bacteria, fungi, oomycetes, and viruses, it can lead to the accumulation of **384**, thereby enhancing their resistance to diseases [123]. So, compound **384** in *B. gymnorrhiza* plants may serve as a crucial participant in the plant’s chemical defense strategy against various pathogen invasions, contributing to the plant’s ability to resist diseases.

### 2.9. Aromatic Compounds

**Figure 15:**
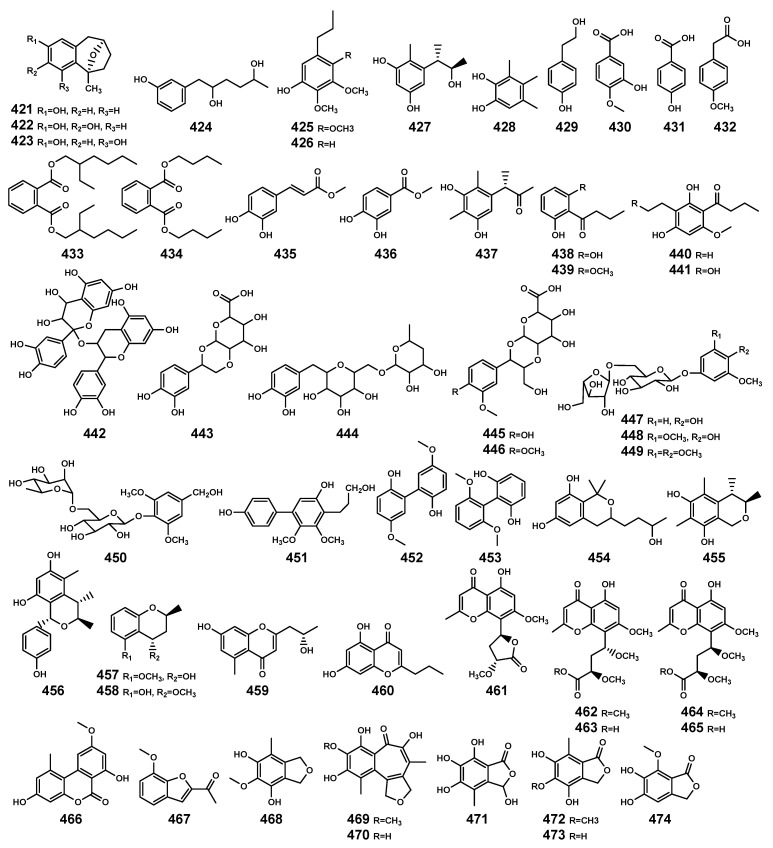
Aromatic compounds (**421–474**) isolated from *Bruguiera* genus plants and their endophytes.

The aromatic compounds (Table 12 and Figure 15) are prevalent in both plants and endophytic fungi secondary metabolites. The majority of the aromatic compounds summarized in this study are phenolic-related aromatic compounds. Previous reports have highlighted the crucial role of phenolic compounds in plants’ defense against various biotic and abiotic stresses [129]. In mangrove plants, some compounds have been identified as substrates of fungal metabolism or (and) signals of plant origin, such as compounds **431**, **435**, and **436**. Additionally, there are other types of aromatic compounds present, including biphenyl derivatives (**451–453**), (iso) benzopyrans (**454–458**), chromones (**459–465**), (iso) benzofurans (**467–468**), and (iso) benzofuranones (**471–474**), among others.

### 2.10. Other Compounds

**Figure 16:**
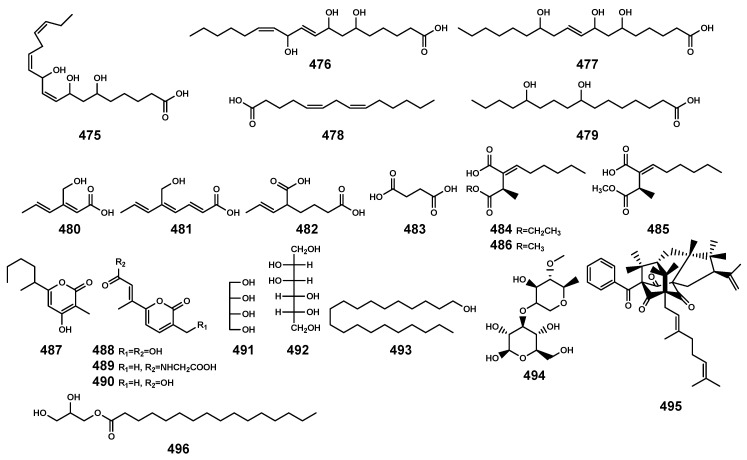
Other compounds (**475–496**) isolated from *Bruguiera* genus plants and their endophytes.

Apart from the previously mentioned compounds, fatty acids (**475*–*486**), 2H-pyran-2-ones (**487*–*490**), alcohols (**491*–*493**), and additional ones (**494*–*496**) were also identified (Table 13 and Figure 16). Specifically, the secondary metabolite **495**, originating from the endophytic fungus *Aspergillus terreus*, was discovered for the first time in the ripe fruits of *Garcinia cowa* [134,135]. It belonged to polyprenylated benzoylphloroglucinols with a unique tetracyclo[7.3.3.3^3,11^.0^3,7^]tetradecane-2,12,14-trione skeleton, and exhibited significant anti-inflammatory and alpha-glucosidase inhibition activities [135].

## 3. Bioactivities

### 3.1. Cytotoxic Activity

Cytotoxic activity stands out as a pharmacological attribute of secondary metabolites derived from *Bruguiera* genus plants and their associated endophytes. The n-butanol extract from *B. gymnorrhiza* showed antitumor activity against A-549 and HL-60 [76]. Methanol extract of *B. gymnorrhiza* displayed selective cytotoxicity against breast ductal carcinoma cells (MDA-MB-435S) with IC_50_ 1.38 mg/mL [137]. Leaves extract of *B. sexangula* demonstrated inhibitory effect on the proliferation of gastric cancer cells in both in vitro and in vivo studies [138]. Qayoom et al. [139] utilized network pharmacology methods and unveiled that brugine (**197**) possesses significant anti-breast cancer activity, exerting its effects through various pathways such as the calcium signaling pathway, cAMP signaling pathway, PI3K-Akt pathway, and others. Additionally, anticancer compounds have been identified in *B. gymnorrhiza* plants and the endophytic fungi of both *B. gymnorrhiza* and *B. sexangula var. rhynchopetala*. The lethality assay using brine shrimp (*Artemia salina* L.) reveals significant cytotoxicity in *B. gymnorrhiza* extracts and certain compounds [9,140]. Table 14 provides a summary of the cytotoxic activity of monomeric compounds. 

### 3.2. Antimicrobial Activity

Previous studies have indicated that crude extracts of *B. gymnorrhiza* exhibit substantial antibacterial activity against a range of bacteria, including the fungal pathogen *Candida albicans* and bacterial pathogens, such as *Micrococcus sp.*, *Staphylococcus aureus* (MTCC 3160), *Klebsiella pneumoniae* (MTCC 4030), *Escherichia coli* (MTCC 42), *E. coli* (NX, AMP, OF resistant strain) [141]. Bibi Sadeer et al. [142] found that ethyl acetate extract of *B. gymnorrhiza* twig, when used in combination with streptomycin and ciprofloxacin, respectively, potentiates their inhibitory effects against MRSA and *P. aeruginosa*. Ethyl acetate extracts obtained from mature leaves, immature leaves, and the bark of *B. sexangula* reveal antibacterial properties against *S. aureus* [143]. Currently, numerous compounds isolated from *B. gymnorrhiza* and endophytes of plants (*B. gymnorrhiza*, *B. sexangula*, *B. sexangula var. rhynchopetala*, and *B. parviflora*) have shown considerable antibacterial activity, as presented in Table 15.

### 3.3. Antioxidant Activity

Studies have revealed significant antioxidant activity in extracts derived from *B. gymnorrhiza*, *B. sexangula*, and *B. cylindrica* [10,144,145]. The antioxidant and polyphenol-rich leaves of *B. gymnorrhiza* can exert hepatoprotective effects by ameliorating liver tissue injury [7]. Condensed tannins from *B. gymnorrhiza* possessed notable anti-tyrosinase and antioxidant capabilities, effectively inhibiting browning reactions in fresh-cut lotus roots [146]. Ethanol extract of *B. sexangula* leaves had antioxidant and melanin inhibition activities without skin irritation [145]. In the in vitro antioxidant activity studies, compounds **469**, **470**, **471**, **472**, **473**, **468**, and **328** showed DPPH radical scavenging activity with IC_50_ values of 57.6, 26.5, 29.3, 85.2, 16.5, 53.1, and 14.7 µM, and ABTS radical scavenging activity with IC_50_ values of 46.4, 29.2, 23.7, 43.1, 23.3, 24.0, and 18.8 µM, respectively [113]. Yao et al. [121] conducted the cellular antioxidant assay and identified that compound **381** exhibited antioxidant activity with an EC_50_ value of 11.79 μM. Compound **382** exhibited effective neuroprotective activity against 1-methyl-4-phenylpyridinium-induced oxidative damage in PC12 cells, and its mechanism involved inhibiting apoptosis in PC12 cells through the mitochondrial pathway [117]. It also attenuated oxidative stress and inflammatory responses induced by lipoteichoic acid in embryonic rat heart cells (H9c2) [147].

### 3.4. Anti-Inflammatory Activity

Studies have indicated the anti-inflammatory activity in extracts from plants within the *Bruguiera* genus, such as *B. gymnorrhiza*, *B. sexangula*, and *B. parviflora*, as well as endophytic fungi associated with *B. gymnorrhiza*. Chen et al. [148] conducted research revealing that aqueous extract of *B. gymnorrhiza* leaves can alleviate dextran sulfate sodium (DSS)-induced ulcerative colitis by inhibiting of NF-*κ*B activation and modulating the gut microbiota. Subsequently, Lin et al. [149] observed that aqueous extracts from *B. gymnorrhiza* fruit also exhibited a protective effect against DSS-induced ulcerative colitis, and the mechanism may be associated with the attenuation of inflammation, activation of the Keap1/Nrf2 signaling pathway, and modulation of the gut microbiota. Furthermore, Zhang et al. [150] reported that methanol extracts of *B. gymnorrhiza* fruits mitigate inflammation in gastric injury by activating the NF-*κ*B pathway, thus conferring gastroprotective effects. Moreover, consumption of *B. gymnorrhiza* fruits has been shown to ameliorate systemic inflammations in obesity, increase circulating satiety hormones, reduce lipid profiles, elevate short-chain fatty acids levels, and promote weight loss [151]. Eldeen et al. [152] discovered that metabolites obtained from *B. cylindrica* possessed inhibitory effects on pro-inflammatory enzymes, including 5-lipoxygenase, cyclooxygenase, and acetylcholinesterase, and on the growth of an induced rheumatoid arthritis synovial fibroblasts. According to reports, compound **367** may serve as the potential primary anti-inflammatory substance in *B. gymnorrhiza* leaves through various possible mechanisms, such as regulation of oxidative stress, suppression of arachidonic acid metabolism, and downregulation of pro-inflammatory cytokines by inhibiting NF-*κ*B [120]. Ukwatta et al. [101,135] employed a cell-based assay for THP-1 cytokine-release assay to quantify the anti-inflammatory activity of compounds **242** and **495**, showcasing IC_50_ values of 6.2 μM and 12.1 µg/mL, respectively. Furthermore, compounds **368**, **369**, **370**, **378**, and **371** from *B. parviflora* leaves exhibited significant inhibitory effects on the inflammatory response of the RAW 264.7 cells induced by lipopolysaccharide with a range of NO production between 11.77 and 13.92 μM, at the concentration of 100 μg/mL [52].

### 3.5. Antiviral Activity

Hou et al. [153]detected that endophytic fungal strains (GXIMD07366, GXIMD07616, GXIMD07384, GXIMD07550, GXIMD07445X) from *B. gymnorrhiza* have anti-hepatitis B virus (HBV) activity. At a concentration of 125 µg/mL, their extracts significantly reduced the HBV-DNA levels in the supernatant of HepG2.2.15 cells, and the inhibition rates were 51%, 47%, 63%, 52%, and 47%, respectively. Compounds **212*–*218** derived from the hypocotyls of *B. gymnorrhiza* displayed moderate anti-HBV activity [91]. Furthermore, the compound **186** exhibited anti-HBV activity, with IC_50_ values of 4.37 mmol/L for HbsAg and 4.89 mmol/L for HBeAg, and therapeutic indices of 2.68 and 2.40 [85]. In addition to anti-HBV activity, compound **44** showed selective anti-HIV activity [30]. It is worth noting that the compound **356** demonstrated moderate antiviral activity against the pathogen SARS-CoV-2, the causative agent of COVID-19, with an EC_50_ value of 29.0 µM [112]. Aqueous extracts from the roots and fruits of *B. gymnorrhiza* inhibited Zika virus (ZIKV) infection at non-cytotoxic concentrations [154]. Moreover, *B. cylindrica*-synthesized AgNP, at a concentration of 30 μg/mL, significantly suppressed the production of dengue viral E protein and downregulated the expression of dengue viral E gene in Vero cells [155].

### 3.6. Antidiabetic Activity

The decoctions of both roots and leaves of *B. gymnorrhiza* have demonstrated varying degrees of antidiabetic activity, ranging from low to moderate efficacy [156]. *B. gymnorrhiza* leaf extracts displayed inhibitory effects on *α*-glucosidase, with an IC_50_ value of 2.670 mg/mL [144]. Extracts derived from *B. cylindrica* leaves contained antidiabetic components, and the ethanol extract of which was considered potential sources of novel bioactive compounds for treating type 2 diabetes [157,158]. Notably, several compounds, including **108**, **117**, **107**, **368**, **369**, **370**, **378**, **371**, and **467** exhibited significant *α*-glucosidase inhibitory activity, with IC_50_ values ranging from 1.1 to 98.0 μg/mL (better than the positive control acarbose) [52,103]. Compounds **242**, **256**, and **495** also show *α*-glucosidase inhibitory activity, with IC_50_ values of 6.9 μM, 5.7 μg/mL, and 7.8 μM, respectively [101,103,135]. Additionally, through in vitro bioactivity assays, it was found that compounds **177** and **174** exhibited significant inhibitory activity against the target molecule associated with type II diabetes, human protein tyrosine phosphatase 1B (PTP1B), with IC_50_ values of 14.9 and 17.6 μM, respectively [72,80,159]. 

### 3.7. Insecticidal and Mosquito Repellent Activity

Currently, in the *Bruguiera* genus and its endophytes, researchers have primarily investigated the anti-plasmodial, anti-*Caenorhabditis elegans*, and mosquito-repellent activities. Bai et al. [61] identified insecticidal activity in meroterpenoids and isocoumarin compounds obtained from the fungus *Penicillium sp*. TGM112, isolated from *B. sexangula var. rhynchopetala*. Compounds **133**, **134**, **135**, **137**, **140**, **141**, **143**, **394**, **395**, **398**, **399**, and **401** showed insecticidal activity against newly hatched larvae of *Helicoverpa armigera* Hubner, with IC_50_ values ranging from 50 to 200 μg/mL [61,62]. Compounds **133**, **134**, **135**, **136**, **137**, **138**, and **139** displayed anti-*C. elegans* activity, with EC_50_ values ranging from 9.4 to 38.2 μg/mL [61]. These types of compounds are expected to be potential candidates for effective and low-toxicity novel biopesticides [62]. The leaf and hypocotyl extracts of *B. cylindrica* exhibited in vitro antiplasmodial activities, with IC_50_ values of 173.75 and 74.81 μg/mL, respectively [160,161]. And compound **110** showed anti-malarial activity, with an EC_50_ value of 8.6 mg/mL [54]. Compound **364** significantly reduced the lifespan of *C. elegans* [118]. Compound **479** displays good nematocidal activity [136]. Compound **495** had anti-filarial activity, with MIC, IC_50_, and LC_50_ values of 0.358, 0.708, and 3.89 mg/mL, respectively [135]. Murugan et al. [155] discovered that *B. cylindrica*-synthesized AgNP had mosquito larvicidal properties and effectively reduced the populations of *Aedes aegypti* larvae and pupae when applied at low doses. 

### 3.8. Enzyme Inhibitory Activity

Homhual et al. [46,79] discovered that in stably transfected HepG2 cells, compounds **91*–*93** and **172*–*174** from the flowers of *B. gymnorrhiza* activated antioxidant response elements (ARE) luciferase activity, with respective EC_50_ values were 7.8, 9.4, 15.7, 3.7, 1.8, and 56.7 µM. Compounds **91**, **172**, and **173** were found to inhibit phorbol ester-induced NF-*κ*B luciferase activity, with IC_50_ values of 1.4, 85.0, and 14.5 μM, respectively [46,79]. Compounds **91** and **172** also exhibited inhibitory effects on cyclooxygenase-2 (COX-2) activity, with IC_50_ values of 0.37 and 6.1 μM, respectively [46,79]. In vitro bioactivity tests revealed that compounds **126**, **129**, **130**, **163**, **226**, **250**, and **251** significantly inhibited *α*-acetylcholinesterase (AChE) with IC_50_ values of 84.26, 5.28, 12.00, 1.89, 3.09, 2.01, and 6.71 µM, respectively [58,69]. Compound **348** demonstrated potent activity against acetylcholinesterase, with an IC_50_ value of 40.26 µM [114].

### 3.9. Other Activities

In addition to the aforementioned biological activities, it has also been reported to possess analgesic, anti-diarrheal, anti-hemolytic activities, and various other pharmacological effects.

The extracts from *B. gymnorrhiza* stem, leaf, and hypocotyl have demonstrated significant analgesic and anti-diarrheal effects [144,162]. When administered at doses of 250 and 500 mg/kg body weight, the leaf and hypocotyl extracts exhibited a remarkable inhibitory effect on castor oil-induced diarrhea mice [144]. Moreover, *B. cylindrica* extracts have shown the ability to inhibit H_2_O_2_-induced hemolysis of bovine erythrocytes [10]. The methanol extract of *B. gymnorrhiza* also exhibited anti-hemolytic activity with an IC_50_ value of 311.28 μg/mL [9]. Extracts from *B. gymnorrhiza*, prepared using different solvents (n-hexane, ethyl acetate, n-butanol, and an aqueous phase), and from leaves of different ages (senescent, mature, and young leaves), exhibited significant inhibitory effects on algae growth [34,163].

## 4. Interactions between *Bruguiera* Genus and Its Endophytes

Plants constitute a complex ecological community, engaging in symbiotic relationships with endophytic fungi, wherein they coexist and evolve together over an extended period [164]. Recent study has reported that the endophytic *Streptomyces parvulus* VCCM 22513 from *B. gymnorrhiza* exhibited significant adaptive responses to abiotic environmental stressors, including antioxidation, salt tolerance, and degradation of aromatic compounds [165]. Moreover, through the review of the literature, it has been observed that both the *Bruguiera* genus and its associated endophytes produce similar or closely related secondary metabolites, including cholesterol (**153**), cytochalasin D (**296**), zygosporin D (**297**), and dibutylphthalate (**434**). This phenomenon may be attributed to the fact that the plants flourish in high-salinity and waterlogged environments. In response to the complex environmental stresses, endophytes associated with *Bruguiera* genus have evolved similar signaling pathways to their host counterparts, enabling information exchange [164,166,167]. This allows the endophytes to elicit defense responses akin to those of the *Bruguiera* genus plants, consequently, synthesize the shared metabolites. 

In the genus *Bruguiera* and its endophytic fungi, cytochalasins (**296*–*314**) were isolated from various sources, including *B. gymnorrhiza* (**296*–*297**), endophytic fungus *Xylaria arbuscula* GZS74 from *B. gymnorrhiza* (**296*–*297**, **300*–*314**), endophytic fungus *Daldinia eschscholtzii* HJ001 from *B. sexangula var. rhynchopetala* (**298*–*299**), and endophytic fungus *Xylaria cubensis* PSU-MA34 from *B. parviflora* (**296**). Cytochalasins are typically derived from the *Xylaria* genus of endophytic fungi [168]. Structurally, they consist of a highly substituted isoindolone ring, featuring a benzyl group at C-3 and fused to an 11- to 14-membered macrocyclic ring [169]. According to their structure–activity relationship, one of the significant toxic structural features of cytochalasins is the presence of a complete perihydroisoindolyl-1-one motif fused to either a [11] or a [13] carbocyclic or a [14] lactone macrocycle ring [169]. Additionally, the C7-OH group may also exhibit toxicity depending on the crop species, sensitivity, and type of cytochalasins [169]. Compound **296** exhibits inhibitory activity against plant pathogens such as *Botrytis cinerea* (at a concentration of 25 μg/disc) [170], *Cladosporium cladosporioides* (at a concentration of 10 μg/mL) [171], *C. sphaerospermum* (at a concentration of 25 μg/mL) [171], and *C. gloeosporioides* (MIC value of 2.46 μmol/mL) [168]. Compound **297** effectively inhibits the shoot elongation of rice seedlings [172]. Compounds **307** and **314** both display nonspecific moderate phytotoxicity against monocotyledonous plant bentgrass and dicotyledonous plant lettuce [173]. Evidently, endophytic fungi of the *Bruguiera* genus aid in plant growth competition and defense against plant pathogens by producing cytochalasins with phytotoxic and antifungal activities, thereby providing protection for *Bruguiera* species’ growth.

Simultaneously, existing research confirms that dibutylphthalate (**434**), a secondary metabolite derived from the *B. gymnorrhiza* plant and its endophytic fungus *Penicillium thomi*, also serves as the primary active metabolite of endophyte *Bacillus sp.* KL5 from the plant *Rumex dentatus*, exhibits significant antagonistic activity against plant pathogens *Fusarium oxysporum* and *Verticillium dahliae*, making it a promising candidate for the prevention and control of postharvest diseases Fusarium root rot in potato [174]. Compound **434** may therefore also contribute to bolstering the *B. gymnorrhiza* plant’s resilience against plant pathogens.

In addition to the shared compounds mentioned above, specific secondary metabolites have been identified in *Bruguiera* genus plants and their endophytes that play crucial roles in promoting plant growth and development, as well as enhancing the plant’s resistance to both biotic and abiotic stresses, like 2,6-dimethoxy-1,4-benzoquinone (**230**), scopoletin (**384**), erythritol (**491**), and mannitol (**492**).

According to the research analysis by Laohavisit et al. [95], quinone molecules may serve as pathogen or danger-associated molecular patterns, such as 2,6-dimethoxy-1,4-benzoquinone (**230**). This quinone signal is perceived by leucine-rich-repeat receptor-like kinases, triggering the expression of defense-related genes and immune responses against bacterial pathogens [95]. Compound **230** produced by host plants can also act as an inducing factor for the development of root parasitic plant absorbers, potentially playing a regulatory role during the parasitic process of these plants [175].

Scopoletin (**384**), as a crucial component in the plant’s natural immune response, plays a role in resisting the invasion of pathogenic microorganisms and promoting the proliferation of beneficial microbes [123]. In healthy plant leaves, due to the instability and toxicity of scopoletin, it is converted into the glycosylated form, scopolin, by glucosyltransferases, then transferred intracellularly and stored in vacuoles, with their accumulation typically maintained at low levels [123]. Upon activation of the leaf defense system by pathogens (fungi, bacteria, and viruses, etc.) or elicitors (flg22, MYB15, MPK3, etc.), scopolin is released from the vacuoles in infected tissues [123,176]. It is then converted to scopoletin by *β*-glucosidases, leading to an increase in scopoletin production [176]. This, in turn, exhibits antimicrobial activity and clears H_2_O_2_ in infected tissues to prevent cell death [176]. The level of disease resistance is correlated with the extent and timing of scopoletin accumulation [177]. The toxic effects of scopoletin may be attributed to the presence of methoxy (-OCH_3_) and hydroxy (-OH) groups on the benzene ring [123]. Upon glycosylation, scopoletin is confined by the cell wall, limiting its toxicity [178]. Depending on the environmental conditions, scopolin and scopoletin in tissues of *B. gymnorrhiza* plant can mutually convert within cells, thereby regulating the level of scopoletin to aid in resisting pathogen invasion.

The compound erythritol (**491**) with antibacterial activity, is one of the main chemical components of the endophytic fungus *Lasiodiplodia pseudotheobromae* APR5 [179], and it is also produced by the endophytic fungus *P. citrinum* ZD6 within the stems of the Chinese medicinal plant *B. gymnorrhiza* [92]. The endophytic fungus *L. pseudotheobromae* APR5, originating from the host plant *Andrographis paniculata*, effectively inhibits the growth of plant pathogenic fungi [179]. Moreover, it produces the plant hormone IAA (indole-3-acetic acid) and iron carrier, promoting the growth and development of the host plant and enhancing its resistance to adverse environmental conditions [179]. Furthermore, the genus *Penicillium* has been confirmed to enhance the resistance of host plants to both biotic and abiotic stresses [180]. As a major member of endophytic fungi within the *Bruguiera* genus, *Penicillium* (26.3%) undoubtedly plays a crucial role in conferring resistance to pathogenic fungal invasion in *Bruguiera* genus plants. 

Mannitol (**492**) is present in *B. gymnorrhiza* plant and its endophytic fungus *P. citrinum* ZD6 [92], serving as both an osmoprotectant and an antioxidant against oxidative stress [181]. However, research suggests that fungal pathogens also secrete mannitol, playing a role in fungal pathogenicity [181]. Mannitol can protect fungal pathogens against plant defense mechanisms based on reactive oxygen [182]. Given the complexity of the existing interactions, the specific mechanisms underlying these processes have not been elucidated as of now.

In summary, utilizing secondary metabolites as a starting point for research, exploring the interactive relationships between the *Bruguiera* genus plants and their endophytic fungi presents an intriguing direction. To delve deeper into the mechanisms underlying the interaction between *Bruguiera* plants and their endophytic fungi, modern “omics” tools, including genome sequencing, comparative genomics, microarrays, next-generation sequencing, metagenomics, metatranscriptomics, and others, can be integrated with various systems biology techniques to explore the role of endophytic fungi in the ecology of *Bruguiera* plants [183].

## 5. Conclusions

The *Bruguiera* genus encompasses a diverse range of plant species housing a wide array of endophytic fungal communities. These organisms are prolific producers of secondary metabolites, which manifest extensive pharmacological effects. Many compounds derived from these sources have demonstrated notable biological activities. Despite the discovery of numerous pharmacological effects, a more comprehensive investigation into the underlying mechanisms is still imperative. The presence of certain compounds in both plants and their endophytes suggests the potential for further exploration into the interaction mechanisms between *Bruguiera* plants and their endophytic fungi. Future research endeavors can be directed towards developing highly active lead compounds with therapeutic potential from the *Bruguiera* genus and its endophytes. 

## Figures and Tables

**Table 2:** Diterpenoids isolated from *Bruguiera* genus plants and their endophytes.

No.	Compound	Source	Reference
59	Gibbererllin A3	*B. gymnorrhiza*, fruit	[39]
60	Gibbererllin A4	*B. gymnorrhiza*, fruit	[39]
61	Gibbererllin A7	*B. gymnorrhiza*, fruit	[39]
62	Steviol	*B. gymnorrhiza*, root bark	[40]
63	Methyl-*ent*-kaur-9(11)-en-13,17-epoxy-16-hydroxy-19-oate	*B. gymnorrhiza*, root bark; *B. sexangula var. rhynchopetala*, stem	[40,41]
64	*ent*-kaur-16-en-13-hydroxy-19-al	*B. gymnorrhiza*, root bark and stem	[40,42]
65	*ent*-kaur-16-en-13,19-diol	*B. gymnorrhiza*, root bark and stem	[40,42]
66	13,16*α*,17-trihydroxy-*ent*-9(11)-kaurene-19-oic acid	*B. gymnorrhiza*, stem	[42]
67	16*α*,17-dihydroxy-*ent*-9(11)-kaurene-19-al	*B. gymnorrhiza*, stem; *B. sexangula var. rhynchopetala*, stem	[41,42]
68	17-chloro-13,16*β*-dihydroxy-*ent*-kauran-19-al	*B. gymnorrhiza*, stem	[42]
69	Methyl-16*α*,17-dihydroxy-*ent*-kauran-19-oate	*B. gymnorrhiza*, stem	[42]
70	16*α*,17-dihydroxy-*ent*-9(11)-kauren-19-oic acid	*B. gymnorrhiza*, stem	[42]
71	Methyl-16*α*,17-dihydroxy-*ent*-9(11)-kauren-19-oate	*B. gymnorrhiza*, stem; *B. sexangula var. rhynchopetala*, stem	[41,42]
72	16*α*,17-dihydroxy-*ent*-kauran-19-al	*B. gymnorrhiza*, stem	[42]
73	16*αH*-17-hydroxy-*ent*-kauran-19-oic acid	*B. gymnorrhiza*, stem	[42]
74	16*αH*-17,19-*ent*-kaurane-diol	*B. gymnorrhiza*, stem	[42]
75	16-*ent*-kauren-19-ol	*B. gymnorrhiza*, stem	[42]
76	(16*R*)-13,17-epoxy-16-hydroxy-*ent*-kaur-9(11)-en-19-al	*B. sexangula var. rhynchopetala*, stem	[41]
77	16,17-dihydroxy-19-nor-*ent*-kaur-9(11)-en-3-one	*B. sexangula var. rhynchopetala*, stem	[41]
78	Ceriopsin F	*B. sexangula var. rhynchopetala*, stem	[41]
79	1*β*,15(*R*)-*ent*-pimar-8(14)-en-1,15,16-triol	*B. gymnorrhiza*, root bark and stem; *B. sexangula var. rhynchopetala*, stem	[40,41,43]
80	*ent*-8(14)-pimarene-15*R*,16-diol	*B. gymnorrhiza*, stem	[43]
81	*ent*-8(14)-pimarene-1*α*,15*R*,16-triol	*B. gymnorrhiza*, stem	[43]
82	(5*R*,9*S*,10*R*,13*S*,15*S*)-*ent*-8(14)-pimarene-1-oxo-15*R*,16-diol	*B. gymnorrhiza*, stem	[43]
83	15(*S*)-isopimar-7-en-15,16-diol	*B. gymnorrhiza*, root bark and stem	[40,43]
84	Isopimar-7-ene-1*β*, 15*S*, 16-triol	*B. gymnorrhiza*, stem	[43]
85	(4*R*,5*S*,8*R*,9*R*,10*S*,13*S*)-*ent*-17-hydroxy-16-oxobeyeran-19-al	*B. gymnorrhiza*, stem; *B. sexangula var. rhynchopetala*, stem	[41,42]
86	17-hydroxy-16-oxobeyer-9(11)-en-19-al	*B. sexangula var. rhynchopetala*, stem	[41]

**Table 3:** Triterpenoids isolated from *Bruguiera* genus plants and their endophytes.

No.	Compound	Source	Reference
91	Bruguierin A	*B. gymnorrhiza*, flower	[46]
92	Bruguierin B	*B. gymnorrhiza*, flower	[46]
93	Bruguierin C	*B. gymnorrhiza*, flower	[46]
94	Sexangulic acid	*B. sexangula*, stem	[47]
95	11-oxo-12*α*-acetoxy-4,4-dimethyl-24-methylene-5*α*-cholesta-8,14-diene-2*α*,3*β*-diol	*Penicillium sp.* J41221 (*B. sexangula var. rhynchopetala*, endophytic fungus)	[48]
96	12*α*-acetoxy-4,4-dimethyl-24-methylene-5*α*-cholesta-8-momoene-3*β*,11*β*-diol	*Penicillium sp.* J41221 (*B. sexangula var. rhynchopetala*, endophytic fungus)	[48]
97	12*α*-acetoxy-4,4-dimethyl-24-methylene-5*α*-cholesta-8,14-diene-3*β*,11*β*-diol	*Penicillium sp.* J41221 (*B. sexangula var. rhynchopetala*, endophytic fungus)	[48]
98	12*α*-acetoxy-4,4-dimethyl-24-methylene-5*α*-cholesta-8,14-diene-2*α*,3*β*,11*β*-triol	*Penicillium sp.* J41221 (*B. sexangula var. rhynchopetala*, endophytic fungus)	[48]
99	Gymnorhizol	*B. gymnorrhiza*, stem and leaf	[49]
100	*β*-amyrin	*B. gymnorrhiza*, leaf	[50]
101	Oleanolic acid	*B. gymnorrhiza*, leaf	[50]
102	(15*α*)-15-hydroxysoyasapogenol B	*Pestalotiopsis clavispora* (*B. sexangula*, endophytic fungus)	[51]
103	(7*β*,15*α*)-7,15-dihydroxysoyasapogenol B	*P. clavispora* (*B. sexangula*, endophytic fungus)	[51]
104	(7*β*)-7,29-dihydroxysoyasapogenol B	*P. clavispora* (*B. sexangula*, endophytic fungus)	[51]
105	*α*-amyrin	*B. gymnorrhiza*, leaf	[50]
106	Ursolic acid	*B. gymnorrhiza*, leaf;	[50]
107	Corosolic acid	*B. parviflora*, leaf	[52]
108	Lupeol	*B. gymnorrhiza*, stem and leaf *B. cylindrica*, fruit and hypocotyl *B. parviflora*, fruit and leaf	[50,52,53,54,55]
109	Lupenone	*B. gymnorrhiza*, stem and leaf *B. cylindrica*, fruit and hypocotyl *B. parviflora*, fruit	[53,54,55]
110	3-(*Z*)-caffeoyllupeol	*B. parviflora*, fruit	[54]
111	3*β*-*E*-caffeoyllupeol	*B. parviflora*, fruit; *B. cylindrica*, fruit and hypocotyl	[54,55]
112	3*α*-*E*-coumaroyllupeol	*B. cylindrica*, fruit and hypocotyl	[55]
113	3*α*-*Z*-coumaroyllupeol	*B. cylindrica*, fruit and hypocotyl	[55]
114	3*β*-*E*-coumaroyllupeol	*B. parviflora*, fruit; *B. cylindrica*, fruit and hypocotyl	[54,55]
115	3*β*-*Z*-coumaroyllupeol	*B. cylindrica*, fruit and hypocotyl	[55]
116	3*α*-lupeol	*B. cylindrica*, fruit and hypocotyl	[55]
117	Betulinic acid	*B. parviflora*, leaf	[52]
118	3*α*-feruloyltaraxerol dichloromethane solvate	*B. cylindrica*, fruit	[56]
119	3*α*-*E*-feruloyltaraxerol	*B. cylindrica*, fruit	[57]
120	3*α*-*Z*-feruloyltaraxerol	*B. cylindrica*, fruit	[57]
121	3*β*-*E*-feruloyltaraxerol	*B. cylindrica*, fruit	[57]
122	3*β*-*Z*-feruloyltaraxerol	*B. cylindrica*, fruit	[57]
123	3*α*-*E*-coumaroyltaraxerol	*B. cylindrica*, fruit	[57]
124	3*α*-*Z*-coumaroyltaraxerol	*B. cylindrica*, fruit	[57]
125	3*α*-taraxerol	*B. cylindrica*, fruit	[57]
126	3*β*-taraxerol	*B. cylindrica*, fruit and leaf	[57,58]
127	14-taraxeren-3-one	*B. gymnorrhiza*, stem and leaf	[53]
128	3*α*-*E*-caffeoyltaraxerol	*B. cylindrica*, fruit and hypocotyl	[55]
129	3*β*-(*Z*)-coumaroyltaraxerol	*B. cylindrica*, leaf	[58]
130	3*β*-(*E*)-coumaroyltaraxerol	*B. cylindrica*, leaf	[58]

**Table 4:** Meroterpenoids isolated from *Bruguiera* genus plants and their endophytes.

No.	Compound	Source	Reference
131	Dehydroaustin	*Penicillium citrinum* HL-5126 (the leaf of *B. sexangula var. rhynchopetala*, endophytic fungus)	[60]
132	11*β*-acetoxyisoaustinone	*P. citrinum* HL-5126 (the leaf of *B. sexangula var. rhynchopetala*, endophytic fungus) *Penicillium sp.* TGM112 (*B. sexangula var. rhynchopetala*, fungus);	[60,61]
133	Austinol	*P. citrinum* HL-5126 (the leaf of *B. sexangula var. rhynchopetala*, endophytic fungus); *Penicillium sp.* TGM112 (*B. sexangula var. rhynchopetala*, fungus)	[60,61]
134	Penicianstinoid A	*Penicillium sp.* TGM112 (*B. sexangula var. rhynchopetala*, fungus)	[61]
135	Penicianstinoid B	*Penicillium sp.* TGM112 (*B. sexangula var. rhynchopetala*, fungus)	[61]
136	Furanoaustinol	*Penicillium sp.* TGM112 (*B. sexangula var. rhynchopetala*, fungus)	[61]
137	1,2-dihydro-7-hydroxydehydroaustin	*Penicillium sp.* TGM112 (*B. sexangula var. rhynchopetala*, fungus)	[61]
138	7-hydroxydehydroaustin	*Penicillium sp.* TGM112 (*B. sexangula var. rhynchopetala*, fungus)	[61]
139	Dehydroaustinol	*Penicillium sp.* TGM112 (*B. sexangula var. rhynchopetala*, fungus)	[61]
140	Austin	*Penicillium sp.* TGM112 (*B. sexangula var. rhynchopetala*, fungus)	[61]
141	Penicianstinoid C	*Penicillium sp.* TGM112 (*B. sexangula var. rhynchopetala*, fungus)	[62]
142	Penicianstinoid D	*Penicillium sp.* TGM112 (*B. sexangula var. rhynchopetala*, fungus)	[62]
143	Penicianstinoid E	*Penicillium sp.* TGM112 (*B. sexangula var. rhynchopetala*, fungus)	[62]
144	Nectrianolin D	*Clonostachys rosea* B5–2 and *Nectria pseudotrichia* B69–1 (the branch of *B. gymnorrhiza*, endophytic fungus)	[63]
145	Furanocochlioquinol	*C. rosea* B5–2 and *N. pseudotrichia* B69–1 (the branch of *B. gymnorrhiza*, endophytic fungus)	[63]
146	Furanocochlioquinone	*C. rosea* B5–2 and *N. pseudotrichia* B69–1 (the branch of *B. gymnorrhiza*, endophytic fungus)	[63]
147	Nectripenoid B	*C. rosea* B5–2 and *N. pseudotrichia* B69–1 (the branch of *B. gymnorrhiza*, endophytic fungus)	[63]
148	Cochlioquinone D	*C. rosea* B5–2 and *N. pseudotrichia* B69–1 (the branch of *B. gymnorrhiza*, endophytic fungus)	[63]
149	Guignardone A	*Phyllosticta capitalensis* (the hypocotyl of *B. sexangula*, endophytic fungus)	[64]
150	12-hydroxylated guignardone A	*P. capitalensis* (the hypocotyl of *B. sexangula*, endophytic fungus)	[64]
151	Guignardone J	*P. capitalensis* (the hypocotyl of *B. sexangula*, endophytic fungus)	[64]
152	Guignardone M	*P. capitalensis* (the hypocotyl of *B. sexangula*, endophytic fungus)	[64]

**Table 5:** Steroids isolated from *Bruguiera* genus plants and their endophytes.

No.	Compound	Source	Reference
153	Cholesterol	*B. gymnorrhiza*, leaf; *Penicillium thomi* (the root of *B. gymnorrhiza*, endophytic fungus);	[50,66]
154	Stigmasterol	*B. gymnorrhiza*, leaf	[50]
155	Sitosterol	*B. gymnorrhiza*, leaf; *P. thomi* (the root of B. gymnorrhiza, endophytic fungus); *B. cylindrica*, leaf	[50,58,66]
156	Stigmast-7-en-3β-ol	*B. gymnorrhiza*, leaf	[50]
157	*β*-daucosterol	*B. gymnorrhiza*, stem and leaf;	[53]
158	*β*-sitosteryl linoleate	*Phomopsis longicolla* HL-2232 (the leaf of *B. sexangula var. rhynchopetala*, endophytic fungus)	[67]
159	Ergosterol	*Penicillium sclerotiorum* (the inner bark of *B. gymnorrhiza*, endophytic fungus);	[68]
160	Campesterol	*B. gymnorrhiza*, leaf	[50]
161	3*β*,5*α*-dihydroxy-(22*E*,24*R*)-ergosta-7,22-dien-6-one	*A. terreus* No. GX7-3B (the branch of *B. gymnorrhiza*, endophytic fungus)	[69]
162	3*β*,5*α*,14*α*-trihydroxy-(22*E*,24*R*)-ergosta-7,22-dien-6-one	*A. terreus* No. GX7-3B (the branch of *B. gymnorrhiza*, endophytic fungus)	[69]
163	NGA0187	*A. terreus* No. GX7-3B (the branch of *B. gymnorrhiza*, endophytic fungus); *Penicillium sp.* GD6 (the stem bark of *B. gymnorrhiza*, endophytic fungus)	[69,70]
164	11-O-acetyl-NGA0187	*Penicillium sp.* GD6 (the stem bark of *B. gymnorrhiza*, endophytic fungus)	[70]
165	Ergosta-7,22-diene-3*β*,5*α*,6*β*-triol	*P. thomi* (the root of *B. gymnorrhiza*, endophytic fungus); *Penicillium sp.* J41221 (*B. sexangula var. rhynchopetala*, endophytic fungus); *P. longicolla* HL-2232 (the leaf *B. sexangula var. rhynchopetala*, endophytic fungus)	[48,66,67]
166	(3*β*,5*α*,6*β*,22*E*)-6-methoxyergosta-7,22-diene-3,5-diol	*Penicillium sp.* J41221 (*B. sexangula var. rhynchopetala*, endophytic fungus);	[48]
167	(22*E*)-5*α*,8*α*-epidioxyergosta-6,22-dien-3*β*-ol	*P. sclerotiorum* (the inner bark of *B. gymnorrhiza*, endophytic fungus); *P. longicolla* HL-2232 (the leaf *B. sexangula var. rhynchopetala*, endophytic fungus)	[67,68]
168	Cyclocitrinol	*Penicillium sp.* GD6 (the stem bark of *B. gymnorrhiza*, endophytic fungus)	[70]
169	Fortisterol	*P. longicolla* HL-2232 (the leaf *B. sexangula var. rhynchopetala*, endophytic fungus)	[67]

**Table 6:** Sulfides isolated from *Bruguiera* genus plants and their endophytes.

No.	Compound	Source	Reference
170	(-)-3,4-dihydro-3-hydroxy-7-methoxy-2*H*-1,5-benzodithiepine-6,9-dione	*B. sexangula var. rhynchopetala*, stem	[41]
171	1,2-dithiolane	*B. gymnorrhiza*, stem and leaf; *B. cylindrica*, stem and bark	[76,77]
172	Brugierol	*B. gymnorrhiza*, stem, leaf, and flower; *B. sexangula var. rhynchopetala*, stem; *B. cylindrica*, stem and bark	[41,72,76,77,78,79]
173	Isobrugierol	*B. gymnorrhiza*, stem, leaf and flower; *B. sexangula var. rhynchopetala*, stem; *B. cylindrica*, stem and bark	[41,72,76,77,78,79]
174	Bruguiesulfurol	*B. gymnorrhiza*, flower	[72,79,80]
175	Trans-3,3′-dihydroxy-1,5,1′,5′-tetrathiacyclodecane	*B. gymnorrhiza*, stem and leaf	[80]
176	Cis-3,3′-dihydroxy-1,5,1′,5′-tetrathiacyclodecane	*B. gymnorrhiza*, stem and leaf	[80]
177	Gymnorrhizol	*B. gymnorrhiza*, stem, leaf and flower;	[72,76,80]
178	Neogymnorrhizol	*B. gymnorrhiza*, stem, leaf and flower	[72,80]
179	Thiocladospolide A	*C. cladosporioides* MA-299 (the leaf of *B. gymnorrhiza*, endophytic fungus)	[21]
180	Thiocladospolide B	*C. cladosporioides* MA-299 (the leaf of *B. gymnorrhiza*, endophytic fungus)	[21]
181	Thiocladospolide C	*C. cladosporioides* MA-299 (the leaf of *B. gymnorrhiza*, endophytic fungus)	[21]
182	Thiocladospolide D	*C. cladosporioides* MA-299 (the leaf of *B. gymnorrhiza*, endophytic fungus)	[21]
183	Pandangolide 3	*C. cladosporioides* MA-299 (the leaf of *B. gymnorrhiza*, endophytic fungus)	[21]
184	Thiocladospolide F	*C. cladosporioides* MA-299 (the leaf of *B. gymnorrhiza*, endophytic fungus)	[75]
185	Thiocladospolide G	*C. cladosporioides* MA-299 (the leaf of *B. gymnorrhiza*, endophytic fungus)	[75]

**Table 7:** Alkaloids and Peptides isolated from *Bruguiera* genus plants and their endophytes.

No.	Compound	Source	Reference
186	Gymnorrhizin A	*B. gymnorrhiza*, hypocotyl	[85]
187	N-(2-hydroxy-4-methoxyphenyl)acetamide	*P. thomi* (the root of *B. gymnorrhiza*, endophytic fungus)	[66]
188	Antimycin A18	*Streptomyces albidoflavus* 107A-01824 (the leaf of *B. gymnorrhiza*, endophytic actinomycete)	[86]
189	(2*S*,2′*R*,3*R*,4*E*,8*E*,3′*E*)-2-(2′-hydroxy-3′-octadecenoylamino)-9-methyl-4,8-octadecadiene-l,3-diol	*P. longicolla* HL-2232 (the leaf *B. sexangula var. rhynchopetala*, endophytic fungus)	[67]
190	N,N′-diphenyl urea	*P. longicolla* HL-2232 (the leaf *B. sexangula var. rhynchopetala*, endophytic fungus)	[67]
191	(*E*)-tert-butyl(3-cinnamamidopropyl)carbamate	*P. citrinum* HL-5126 (*B. sexangula var. rhynchopetala*, endophytic fungus)	[87]
192	3-(dimethylaminomethyl)-1-(1,1-dimethyl-2-propenyl)indole	*P. sclerotiorum* (the inner bark of *B. gymnorrhiza*, endophytic fungus)	[68]
193	Meleagrin	*Penicillium sp.* GD6 (the stem bark of *B. gymnorrhiza*, endophytic fungus)	[88]
194	Uridine	*P. longicolla* HL-2232 (the leaf *B. sexangula var. rhynchopetala*, endophytic fungus)	[67]
195	6-aminopurine-9-carboxylic acid methyl ester	*P. longicolla* HL-2232 (the leaf *B. sexangula var. rhynchopetala*, endophytic fungus)	[67]
196	Adenine riboside	*P. longicolla* HL-2232 (the leaf *B. sexangula var. rhynchopetala*, endophytic fungus)	[67]
197	Brugine	*B. sexangula*, stem bark; *B. cylindrica*, stem and bark	[11,77,89]
198	Scalusamide A	*Penicillium sp.* GD6 (the stem bark of *B. gymnorrhiza*, endophytic fungus)	[88]
199	Penibruguieramine A	*Penicillium sp.* GD6 (the stem bark of *B. gymnorrhiza*, endophytic fungus)	[88]
200	Anacine	*P. sclerotiorum* (the inner bark of *B. gymnorrhiza*, endophytic fungus)	[68]
201	Aurantiomide C	*P. sclerotiorum* (the inner bark of *B. gymnorrhiza*, endophytic fungus)	[68]
202	Viridicatol	*P. sclerotiorum* (the inner bark of *B. gymnorrhiza*, endophytic fungus)	[68]
203	Viridicatin	*P. sclerotiorum* (the inner bark of *B. gymnorrhiza*, endophytic fungus)	[68]
204	3-O-methylviridicatin	*P. sclerotiorum* (the inner bark of *B. gymnorrhiza*, endophytic fungus)	[68]
205	(+)-Cyclopenol	*P. sclerotiorum* (the inner bark of *B. gymnorrhiza*, endophytic fungus)	[68]
206	Roquefortine F	*Penicillium sp.* GD6 (the stem bark of *B. gymnorrhiza*, endophytic fungus)	[88]
207	Penilloid A	*Penicillium sp.* GD6 (the stem bark of *B. gymnorrhiza*, endophytic fungus)	[90]
208	Cyclo(dehydrohistidyl-L-tryptophyl)	*Penicillium sp.* GD6 (the stem bark of *B. gymnorrhiza*, endophytic fungus)	[90]
209	5*S*-hydroxynorvalines-Ile	*Penicillium sp.* GD6 (the stem bark of *B. gymnorrhiza*, endophytic fungus)	[90]
210	3*S*-hydroxylcyclo(*S*-Pro-*S*-Phe)	*Penicillium sp.* GD6 (the stem bark of *B. gymnorrhiza*, endophytic fungus)	[90]
211	Cyclo(*S*-Phe-*S*-Gln)	*Penicillium sp.* GD6 (the stem bark of *B. gymnorrhiza*, endophytic fungus)	[90]
212	Menisdurin B	*B. gymnorrhiza*, hypocotyl	[91]
213	Menisdurin C	*B. gymnorrhiza*, hypocotyl	[91]
214	Menisdurin D	*B. gymnorrhiza*, hypocotyl	[91]
215	Menisdurin E	*B. gymnorrhiza*, hypocotyl	[91]
216	Menisdurin	*B. gymnorrhiza*, hypocotyl	[91]
217	Coclauril	*B. gymnorrhiza*, hypocotyl	[91]
218	Menisdaurilide	*B. gymnorrhiza*, hypocotyl	[91]
219	Uracil	*P. thomi* (the root of *B. gymnorrhiza*, endophytic fungus)	[66]
220	Cyclo-(Ala-Gly)	*P. thomi* (the root of *B. gymnorrhiza*, endophytic fungus); *Penicillium citrinum* ZD6 (the stem of *B. gymnorrhiza*, endophytic fungus)	[66,92]
221	Cyclo-(Pro-Gly)	*P. thomi* (the root of *B. gymnorrhiza*, endophytic fungus)	[66]
222	Cyclo-(Ala-Pro)	*P. thomi* (the root of *B. gymnorrhiza*, endophytic fungus)	[66]
223	3-benzylpiperazine-2,5-dione	*Gloesporium sp.* (*B. gymnorrhiza*, endophytic fungus)	[93]
224	3-benzyl-6-(4-hydroxybenzyl) piperazine-2,5-dione	*Gloesporium sp.* (*B. gymnorrhiza*, endophytic fungus)	[93]
225	3-(2-methylpropyl)-2,5-piperazinedione	*Gloesporium sp.* (*B. gymnorrhiza*, endophytic fungus)	[93]
226	Beauvericin	*A. terreus* No. GX7-3B (the branch of *B. gymnorrhiza*, endophytic fungus)	[69]
227	5,5′-epoxy-MKN-349A	*Penicillium sp.* GD6 (the stem bark of *B. gymnorrhiza*, endophytic fungus)	[70]
228	Aspergilumamide A	*Aspergillus sp.* 33241 (*B. sexangula var. rhynchopetala*, fungus)	[94]
229	Penilumamide	*Aspergillus sp.* 33241 (*B. sexangula var. rhynchopetala*, fungus)	[94]

**Table 8:** Quinones isolated from *Bruguiera* genus plants and their endophytes.

No.	Compound	Source	Reference
230	2,6-dimethoxy-1,4-benzoquinone	*B. sexangula var. rhynchopetala*, stem	[41]
231	2-chloro-5-methoxy-3-methylcyclohexa-2,5-diene-1,4-dione	*Xylaria cubensis* PSU-MA34 (the branch of *B. parviflora*, endophytic fungus)	[97]
232	Palmarumycins BG1	*B. gymnorrhiza*, stem and leaf	[98]
233	Palmarumycins BG2	*B. gymnorrhiza*, stem and leaf	[98]
234	Palmarumycins BG3	*B. gymnorrhiza*, stem and leaf	[98]
235	Palmarumycins BG4	*B. gymnorrhiza*, stem and leaf	[98]
236	Palmarumycins BG5	*B. gymnorrhiza*, stem and leaf	[98]
237	Palmarumycins BG6	*B. gymnorrhiza*, stem and leaf	[98]
238	Palmarumycins BG7	*B. gymnorrhiza*, stem and leaf	[98]
239	Preussomerin BG1	*B. gymnorrhiza*, stem and leaf	[98]
240	8-methoxy-1-naphthol	*Daldinia eschscholtzii* PSU-STD57 (the leaf of *B. gymnorrhiza*, endophytic fungus)	[99]
241	1,8-dimethoxynaphthalene	*D. eschscholtzii* PSU-STD57 (the leaf of *B. gymnorrhiza*, endophytic fungus); *Daldinia eschscholtzii* HJ001 (*B. sexangula var. rhynchopetala*, endophytic fungus)	[99,100]
242	Nigronatthaphenyl	*Nigrospora sphaerica* (the mature leaf of *B. gymnorrhiza*, endophytic fungus)	[101]
243	(3*S*)-3,8-dihydroxy-6,7-dimethyl-*α*-tetralone	*D. eschscholtzii* PSU-STD57 (the leaf of *B. gymnorrhiza*, endophytic fungus)	[99]
244	Isosclerone	*D. eschscholtzii* PSU-STD57 (the leaf of *B. gymnorrhiza*, endophytic fungus); *X. cubensis* PSU-MA34 (the branch of *B. parviflora*, endophytic fungus)	[97,99]
245	(4*S*)-3,4-dihydro-4,8-dihydroxy-l(2*H*)-naphthalenoe	*P. citrinum* HL-5126 (the leaf of *B. sexangula var. rhynchopetala*, endophytic fungus)	[102]
246	Regiolone	*P. capitalensis* (the hypocotyl of *B. sexangula*, endophytic fungus)	[64]
247	1,3,8-trimethoxynaphtho[9–c]furan	*D. eschscholtzii* HJ004 (the stem of *B. sexangula var. rhynchopetala*, endophytic fungus)	[103]
248	4-O-methyl eleutherol	*D. eschscholtzii* HJ004 (the stem of *B. sexangula var. rhynchopetala*, endophytic fungus)	[103]
249	8-hydroxy-2-[1-hydroxyethyl]-5,7-dimethoxynaphtho[2,3-b] thiophene-4,9-dione	*A. terreus* No. GX7-3B (the branch of *B. gymnorrhiza*, endophytic fungus)	[69]
250	Anhydrojavanicin	*A. terreus* No. GX7-3B (the branch of *B. gymnorrhiza*, endophytic fungus)	[69]
251	8-O-methylbostrycoidin	*A. terreus* No. GX7-3B (the branch of *B. gymnorrhiza*, endophytic fungus)	[69]
252	8-O-methyljavanicin	*A. terreus* No. GX7-3B (the branch of *B. gymnorrhiza*, endophytic fungus)	[69]
253	Botryosphaerone D	*A. terreus* No. GX7-3B (the branch of *B. gymnorrhiza*, endophytic fungus)	[69]
254	6-ethyl-5-hydroxy-3,7-dimethoxynaphthoquinone	*A. terreus* No. GX7-3B (the branch of *B. gymnorrhiza*, endophytic fungus)	[69]
255	5-hydroxy-2-methoxy-6,7-dimethyl-1,4-naphthoquinone	*D. eschscholtzii* HJ004 (the stem of *B. sexangula var. rhynchopetala*, endophytic fungus)	[103]
256	5-hydroxy-2-methoxynaphtho[9–c] furan-1,4-dione	*D. eschscholtzii* HJ004 (the stem of *B. sexangula var. rhynchopetala*, endophytic fungus)	[103]
257	Incarxanthone A	*Peniophora incarnata* Z4 (*B. gymnorrhiza*, endophytic fungus)	[104]
258	Incarxanthone B	*P. incarnata* Z4 (*B. gymnorrhiza*, endophytic fungus)	[104]
259	Incarxanthone C	*P. incarnata* Z4 (*B. gymnorrhiza*, endophytic fungus)	[104]
260	Incarxanthone D	*P. incarnata* Z4 (*B. gymnorrhiza*, endophytic fungus)	[104]
261	Incarxanthone E	*P. incarnata* Z4 (*B. gymnorrhiza*, endophytic fungus)	[104]
262	Incarxanthone F	*P. incarnata* Z4 (*B. gymnorrhiza*, endophytic fungus)	[104]
263	2,8-Dihydroxyvertixanthone	*P. incarnata* Z4 (*B. gymnorrhiza*, endophytic fungus)	[104]
264	Globosuxanthone B	*P. incarnata* Z4 (*B. gymnorrhiza*, endophytic fungus)	[104]
265	4-chloro-1-hydroxy-3-methoxy-6-methyl-8-methoxycarbonyl-xanthen-9-one	*P. citrinum* HL-5126 (*B. sexangula var. rhynchopetala*, endophytic fungus)	[105]
266	Chloroisosulochrin dehydrate	*P. citrinum* HL-5126 (*B. sexangula var. rhynchopetala*, endophytic fungus)	[105]
267	Emodin	*P. citrinum* ZD6 (the stem of *B. gymnorrhiza*, endophytic fungus)	[92]
268	Auxarthrol C	*Stemphylium sp.* 33231 (*B. sexangula var. rhynchopetala*, endophytic fungus)	[106]
269	Macrosporin-2-O-(6′-acetyl)-*α*-D-glucopyranoside	*Stemphylium sp.* 33231 (*B. sexangula var. rhynchopetala*, endophytic fungus)	[106]
270	Macrosporin	*Stemphylium sp.* 33231 (*B. sexangula var. rhynchopetala*, endophytic fungus)	[106]
271	Macrosporin-7-O-sulfate	*Stemphylium sp.* 33231 (*B. sexangula var. rhynchopetala*, endophytic fungus)	[106]
272	2-O-acetylaltersolanol B	*Stemphylium sp.* 33231 (*B. sexangula var. rhynchopetala*, endophytic fungus)	[106]
273	Altersolanol A	*Stemphylium sp.* 33231 (*B. sexangula var. rhynchopetala*, endophytic fungus)	[106]
274	Altersolanol B	*Stemphylium sp.* 33231 (*B. sexangula var. rhynchopetala*, endophytic fungus); *P. longicolla* HL-2232 (the leaf of *B. sexangula var. rhynchopetala*, endophytic fungus)	[106,107]
275	Altersolanol C	*Stemphylium sp.* 33231 (*B. sexangula var. rhynchopetala*, endophytic fungus)	[106]
276	2-O-acetylaltersolanol L	*Stemphylium sp.* 33231 (*B. sexangula var. rhynchopetala*, endophytic fungus)	[106]
277	Altersolanol L	*Stemphylium sp.* 33231 (*B. sexangula var. rhynchopetala*, endophytic fungus)	[106]
278	Ampelanol	*Stemphylium sp.* 33231 (*B. sexangula var. rhynchopetala*, endophytic fungus)	[106]
279	Tetrahydroaltersolanol B	*Stemphylium sp.* 33231 (*B. sexangula var. rhynchopetala*, endophytic fungus)	[106]
280	Dihydroaltersolanol A	*Stemphylium sp.* 33231 (*B. sexangula var. rhynchopetala*, endophytic fungus)	[106]
281	Alterporriol T	*Stemphylium sp.* 33231 (*B. sexangula var. rhynchopetala*, endophytic fungus)	[106]
282	Alterporriol U	*Stemphylium sp.* 33231 (*B. sexangula var. rhynchopetala*, endophytic fungus)	[106]
283	Alterporriol V	*Stemphylium sp.* 33231 (*B. sexangula var. rhynchopetala*, endophytic fungus)	[106]
284	Alterporriol W	*Stemphylium sp.* 33231 (*B. sexangula var. rhynchopetala*, endophytic fungus)	[106]
285	Alterporriol A	*Stemphylium sp.* 33231 (*B. sexangula var. rhynchopetala*, endophytic fungus)	[106]
286	Alterporriol B	*Stemphylium sp.* 33231 (*B. sexangula var. rhynchopetala*, endophytic fungus)	[106]
287	Alterporriol D	*Stemphylium sp.* 33231 (*B. sexangula var. rhynchopetala*, endophytic fungus)	[106]
288	Alterporriol E	*Stemphylium sp.* 33231 (*B. sexangula var. rhynchopetala*, endophytic fungus)	[106]
289	Alterporriol C	*Stemphylium sp.* 33231 (*B. sexangula var. rhynchopetala*, endophytic fungus)	[106]
290	Alterporriol N	*Stemphylium sp.* 33231 (*B. sexangula var. rhynchopetala*, endophytic fungus)	[106]
291	Alterporriol R	*Stemphylium sp.* 33231 (*B. sexangula var. rhynchopetala*, endophytic fungus)	[106]
292	Alterporriol Q	*Stemphylium sp.* 33231 (*B. sexangula var. rhynchopetala*, endophytic fungus)	[106]
293	2′-acetoxy-7-chlorocitreorosein	*P. citrinum* HL-5126 (*B. sexangula var. rhynchopetala*, endophytic fungus)	[105]
294	Citreorosein	*P. citrinum* HL-5126 (*B. sexangula var. rhynchopetala*, endophytic fungus)	[105]
295	MT-1	*P. citrinum* HL-5126 (*B. sexangula var. rhynchopetala*, endophytic fungus)	[105]

**Table 9:** Polyketides isolated from *Bruguiera* genus plants and their endophytes.

No.	Compound	Source	Reference
296	Cytochalasin D	*B. gymnorrhiza*; *Xylaria arbuscula* GZS74 (the fruit of *B. gymnorrhiza*, endophytic fungus) *X. cubensis* PSU-MA34 (the branch of *B. parviflora*, endophytic fungus)	[35,97,110]
297	Zygosporin D	*B. gymnorrhiza*; *X. arbuscula* GZS74 (the fruit of *B. gymnorrhiza*, endophytic fungus)	[35,110]
298	[11]-cytochalasa-5(6),13-diene-1,21-dione-7,18-dihydroxy-16,18-dimethyl-10-phenyl(7S*,13*E*,16S*,18R*)	*D. eschscholtzii* HJ001 (*B. sexangula var. rhynchopetala*, endophytic fungus)	[100]
299	[11]-cytochalasa-6(12),13-diene-1,21-dione-7,18-dihydroxy16,18-dimethyl-10-phenyl-(7S*,13*E*,16S*,18R*)	*D. eschscholtzii* HJ001 (*B. sexangula var. rhynchopetala*, endophytic fungus)	[100]
300	Arbuschalasins A	*X. arbuscula* GZS74 (the fruit of *B. gymnorrhiza*, endophytic fungus)	[110]
301	Arbuschalasins B	*X. arbuscula* GZS74 (the fruit of *B. gymnorrhiza*, endophytic fungus)	[110]
302	Arbuschalasins C	*X. arbuscula* GZS74 (the fruit of *B. gymnorrhiza*, endophytic fungus)	[110]
303	Arbuschalasins D	*X. arbuscula* GZS74 (the fruit of *B. gymnorrhiza*, endophytic fungus)	[110]
304	Cytochalasin Q	*X. arbuscula* GZS74 (the fruit of *B. gymnorrhiza*, endophytic fungus)	[110]
305	12-hydroxylcytochalasin Q	*X. arbuscula* GZS74 (the fruit of *B. gymnorrhiza*, endophytic fungus)	[110]
306	Cytochalasin D-13,14-epoxid	*X. arbuscula* GZS74 (the fruit of *B. gymnorrhiza*, endophytic fungus)	[110]
307	19,20-epoxycytochalasin D	*X. arbuscula* GZS74 (the fruit of *B. gymnorrhiza*, endophytic fungus)	[110]
308	Cytochalasin O	*X. arbuscula* GZS74 (the fruit of *B. gymnorrhiza*, endophytic fungus)	[110]
309	Cytochalasin P	*X. arbuscula* GZS74 (the fruit of *B. gymnorrhiza*, endophytic fungus)	[110]
310	Cytochalasin C	*X. arbuscula* GZS74 (the fruit of *B. gymnorrhiza*, endophytic fungus)	[110]
311	Deacetylcytochalasin C	*X. arbuscula* GZS74 (the fruit of *B. gymnorrhiza*, endophytic fungus)	[110]
312	6,7-dihydro-7-oxo-cytochalasin C	*X. arbuscula* GZS74 (the fruit of *B. gymnorrhiza*, endophytic fungus)	[110]
313	6,7-dihydro-7-oxo-deacetylcytochalasin C	*X. arbuscula* GZS74 (the fruit of *B. gymnorrhiza*, endophytic fungus)	[110]
314	19, 20-epoxycytochalasin C	*X. arbuscula* GZS74 (the fruit of *B. gymnorrhiza*, endophytic fungus)	[110]
315	1,10-dihydroxy-8-methyldibenz[b, e] oxepin-6,11-dione	No. GX4-1B (the branch of *B. gymnorrhiza*, endophytic fungus)	[111]
316	1,10-dihydroxy-dibenz[b, e]oxepin-6,11-dione	No. GX4-1B (the branch of *B. gymnorrhiza*, endophytic fungus)	[111]
317	2-deoxy-sohirnone C	*Penicillium sp.* GD6 (the stem bark of *B. gymnorrhiza*, endophytic fungus)	[90]
318	Sohirnone A	*Penicillium sp.* GD6 (the stem bark of *B. gymnorrhiza*, endophytic fungus)	[90]
319	3,4-dihydroxybenzoic acid	*P. capitalensis* (the hypocotyl of *B. sexangula*, endophytic fungus)	[64]
320	Penibenzophenone A	*P. citrinum* HL-5126 (*B. sexangula var. rhynchopetala*, endophytic fungus)	[87]
321	Penibenzophenone B	*P. citrinum* HL-5126 (*B. sexangula var. rhynchopetala*, endophytic fungus)	[87]
322	Sulochrin	*P. citrinum* HL-5126 (*B. sexangula var. rhynchopetala*, endophytic fungus)	[87]
323	Sorbicillin	*Trichoderma reesei* SCNU-F0042 (the fresh bark of *B. gymnorrhiza*, endophytic fungus)	[112]
324	Asterric acid	*P. citrinum* HL-5126 (*B. sexangula var. rhynchopetala*, endophytic fungus)	[87]
325	N-butyl asterrate	*P. citrinum* HL-5126 (*B. sexangula var. rhynchopetala*, endophytic fungus)	[87]
326	8-O-methylnodulisporin F	*D. eschscholtzii* HJ004 (the stem of *B. sexangula var. rhynchopetala*, endophytic fungus)	[103]
327	Nodulisporin H	*D. eschscholtzii* HJ004 (the stem of *B. sexangula var. rhynchopetala*, endophytic fungus)	[103]
328	Epicoccolide A	*Epicoccum nigrum* MLY-3 (the leaf of *B. gymnorrhiza*, endophytic fungus)	[113]
329	Divergolide A	*Streptomyces sp.* (*B. gymnorrhiza*, endophytic bacteria)	[22]
330	Divergolide B	*Streptomyces sp.* (*B. gymnorrhiza*, endophytic bacteria)	[22]
331	Divergolide C	*Streptomyces sp.* (*B. gymnorrhiza*, endophytic bacteria)	[22]
332	Divergolide D	*Streptomyces sp.* (*B. gymnorrhiza*, endophytic bacteria)	[22]
333	Divergolide E	*Streptomyces sp.* (*B. gymnorrhiza*, endophytic bacteria)	[22]
334	Divergolide F	*Streptomyces sp.* (*B. gymnorrhiza*, endophytic bacteria)	[22]
335	Divergolide G	*Streptomyces sp.* (*B. gymnorrhiza*, endophytic bacteria)	[22]
336	Divergolide H	*Streptomyces sp.* (*B. gymnorrhiza*, endophytic bacteria)	[22]
337	Seco-patulolide C	*C. cladosporioides* MA-299 (the leaf of *B. gymnorrhiza*, endophytic fungus)	[21]
338	5*R*-hydroxyrecifeiolide	*C. cladosporioides* MA-299 (the leaf of *B. gymnorrhiza*, endophytic fungus)	[114]
339	5*S*-hydroxyrecifeiolide	*C. cladosporioides* MA-299 (the leaf of *B. gymnorrhiza*, endophytic fungus)	[114]
340	Pandangolide 1	*C. cladosporioides* MA-299 (the leaf of *B. gymnorrhiza*, endophytic fungus)	[114]
341	Cladocladosin A	*C. cladosporioides* MA-299 (the leaf of *B. gymnorrhiza*, endophytic fungus)	[75]
342	Verrucosidin	*P. sclerotiorum* (the inner bark of *B. gymnorrhiza*, endophytic fungus)	[68]
343	Acetylchrysopyrone B	*T. reesei* SCNU-F0042 (the fresh bark of *B. gymnorrhiza*, endophytic fungus)	[112]
344	Saturnispol H	*T. reesei* SCNU-F0042 (the fresh bark of *B. gymnorrhiza*, endophytic fungus)	[112]
345	Infectopyrone A	*Stemphylium sp.* 33231 (the leaf *B. sexangula var. rhynchopetala*, endophytic fungus)	[106]
346	Infectopyrone B	*Stemphylium sp.* 33231 (the leaf *B. sexangula var. rhynchopetala*, endophytic fungus)	[106]
347	6,8-dihydroxy-5-methoxy-3-methyl-1*H*-isochromen-1-one	*P. capitalensis* (the hypocotyl of *B. sexangula*, endophytic fungus)	[64]
348	*ent*-cladospolide F	*C. cladosporioides* MA-299 (the leaf of *B. gymnorrhiza*, endophytic fungus)	[114]
349	Cladospolide G	*C. cladosporioides* MA-299 (the leaf of *B. gymnorrhiza*, endophytic fungus)	[114]
350	Cladospolide H	*C. cladosporioides* MA-299 (the leaf of *B. gymnorrhiza*, endophytic fungus)	[114]
351	Iso-cladospolide B	*C. cladosporioides* MA-299 (the leaf of *B. gymnorrhiza*, endophytic fungus)	[114]
352	(-)-dihydrovertinolide	*C. rosea* B5-2 (the branch of *B. gymnorrhiza*, endophytic fungus)	[108]
353	(-)-vertinolide	*C. rosea* B5-2 (the branch of *B. gymnorrhiza*, endophytic fungus)	[108]
354	Xenofuranone B	*P. capitalensis* (the hypocotyl of *B. sexangula*, endophytic fungus)	[64]
355	14-hydroxybislongiquinolide	*T. reesei* SCNU-F0042 (the fresh bark of *B. gymnorrhiza*, endophytic fungus)	[112]
356	20-hydroxybislongiquinolide	*T. reesei* SCNU-F0042 (the fresh bark of *B. gymnorrhiza*, endophytic fungus)	[112]
357	14, 20-dihydroxybislongiquinolide	*T. reesei* SCNU-F0042 (the fresh bark of *B. gymnorrhiza*, endophytic fungus)	[112]
358	Bislongiquinolide	*T. reesei* SCNU-F0042 (the fresh bark of *B. gymnorrhiza*, endophytic fungus)	[112]
359	Trichodimerol	*T. reesei* SCNU-F0042 (the fresh bark of *B. gymnorrhiza*, endophytic fungus)	[112]
360	Bisorbicillinolide	*T. reesei* SCNU-F0042 (the fresh bark of *B. gymnorrhiza*, endophytic fungus)	[112]
361	Saturnispol B	*T. reesei* SCNU-F0042 (the fresh bark of *B. gymnorrhiza*, endophytic fungus)	[112]
362	Bisvertinolone	*T. reesei* SCNU-F0042 (the fresh bark of *B. gymnorrhiza*, endophytic fungus)	[112]
363	Penicitrinone acetate	*Penicillium sp.* B21 (the leaf *B. sexangula var. rhynchopetala*, endophytic fungus)	[115]

**Table 10:** Flavonoids isolated from *Bruguiera* genus plants and their endophytes.

No.	Compound	Source	Reference
364	3′,4′,5′-trihydroxy-7-hydroxy-5-methoxyflavone	*B. gymnorrhiza*, leaf	[118,119]
365	Gramrione	*B. gymnorrhiza*, stem and leaf	[53,119]
366	3′,4′,5,7-tetrahydroxy methylflavone	*B. gymnorrhiza*, stem and leaf	[53]
367	5,7-dihydroxy-2-[3-hydroxy-4,5-dimethoxy-phenyl]-chromen-4-one	*B. gymnorrhiza*, leaf	[120]
368	Taxifolin	*B. parviflora*, leaf	[52]
369	Quercetin	*B. parviflora*, leaf	[52]
370	Myricetin	*B. parviflora*, leaf	[52]
371	Kaempferol	*B. parviflora*, leaf	[52]
372	Luteolin 5-methyl ether 7-O-*β*-D-glucopyranoside	*B. gymnorrhiza*, leaf	[119]
373	7,4′-dihydroxy-5,3′-dimethoxyflavone 7-O-*β*-D-glucopyranoside	*B. gymnorrhiza*, leaf	[119]
374	7,4′,5′-trihydroxy-5,3′-dimethoxyflavone 7-O-*β*-D- glucopyranoside	*B. gymnorrhiza*, leaf	[119]
375	7,4′-dihydroxy-5-methoxyflavone 7-O-*β*-D-glucopyranoside	*B. gymnorrhiza*, leaf	[119]
376	Quercetin-3-O-*β*-D-glucopyranoside	*B. gymnorrhiza*, stem and leaf	[53,119]
377	Astragalin	*B. gymnorrhiza*, stem and leaf	[53]
378	Rutin	*B. gymnorrhiza*, stem and leaf; *B. parviflora*, leaf	[52,53,119]
379	Kaempferol 3-O-rutinoside	*B. gymnorrhiza*, leaf	[119]
380	Myricetin 3-O-rutinoside	*B. gymnorrhiza*, leaf	[119]
381	Brugymnoside A	*B. gymnorrhiza*, hypocotyl	[121]
382	(*Z*)-7,4’-dimethoxy-6-hydroxy-aurone-4-O-*β*-glucopyranoside	*P. citrinum* (*B. gymnorrhiza*, endophytic fungus)	[117]

**Table 11:** Phenylpropanoids isolated from *Bruguiera* genus plants and their endophytes.

No.	Compound	Source	Reference
383	3-phenylpropanoic acid	*Gloesporium sp.* (*B. gymnorrhiza*, endophytic fungus)	[93]
384	Scopoletin	*B. gymnorrhiza*, hypocotyl	[124]
385	3-hydroxymethyl-6,8-dimethoxycoumarin	No. GX4-1B (the branch of *B. gymnorrhiza*, endophytic fungus)	[111]
386	6-hydroxy-4-hydroxymethyl-8-methoxy-3- methylisocoumarin	No. GX4-1B (the branch of *B. gymnorrhiza*, endophytic fungus)	[111]
387	(3R*,4S*)-6,8-dihydroxy-3,4,7-trimethylisocoumarin	*Penicillium sp.* 091402 (the root of *B. sexangula*, endophytic fungus)	[125]
388	(3*R*,4*S*)-6,8-dihydroxy-3,4,5-trimethylisocoumarin	*Penicillium sp.* 091402 (the root of *B. sexangula*, endophytic fungus)	[125]
389	€-6-hydroxymellein	*P. citrinum* HL-5126 (the leaf of *B. sexangula var. rhynchopetala*, endophytic fungus	[102]
390	Penicimarin G	*P. citrinum* HL-5126 (the leaf of *B. sexangula var. rhynchopetala*, endophytic fungus	[60]
391	Penicimarin H	*P. citrinum* HL-5126 (the leaf of *B. sexangula var. rhynchopetala*, endophytic fungus	[60]
392	Penicimarin I	*P. citrinum* HL-5126 (the leaf of *B. sexangula var. rhynchopetala*, endophytic fungus	[60]
393	Aspergillumarin A	*P. citrinum* HL-5126 (the leaf of *B. sexangula var. rhynchopetala*, endophytic fungus; *Penicillium sp.* TGM112 (*B. sexangula var. rhynchopetala*, fungus)	[60,61]
394	Peniciisocoumarin A	*Penicillium sp.* TGM112 (*B. sexangula var. rhynchopetala*, fungus)	[61]
395	Peniciisocoumarin B	*Penicillium sp.* TGM112 (*B. sexangula var. rhynchopetala*, fungus)	[61]
396	Peniciisocoumarin C	*Penicillium sp.* TGM112 (*B. sexangula var. rhynchopetala*, fungus)	[61]
397	Peniciisocoumarin D	*Penicillium sp.* TGM112 (*B. sexangula var. rhynchopetala*, fungus)	[61]
398	Peniciisocoumarin E	*Penicillium sp.* TGM112 (*B. sexangula var. rhynchopetala*, fungus)	[61]
399	Peniciisocoumarin F	*Penicillium sp.* TGM112 (*B. sexangula var. rhynchopetala*, fungus)	[61]
400	Peniciisocoumarin G	*Penicillium sp.* TGM112 (*B. sexangula var. rhynchopetala*, fungus)	[61]
401	Peniciisocoumarin H	*Penicillium sp.* TGM112 (*B. sexangula var. rhynchopetala*, fungus)	[61]
402	(*R*)-3-(3-hydroxypropyl)-8-hydroxy-3,4-dihydroisocoumarin	*Penicillium sp.* TGM112 (*B. sexangula var. rhynchopetala*, fungus)	[61]
403	Penicimarin C	*Penicillium sp.* TGM112 (*B. sexangula var. rhynchopetala*, fungus)	[61]
404	(-)-Orthosporin	*D. eschscholtzii* HJ004 (the stem of *B. sexangula var. rhynchopetala*, endophytic fungus)	[103]
405	Diaporthin	*D. eschscholtzii* HJ004 (the stem of *B. sexangula var. rhynchopetala*, endophytic fungus)	[103]
406	6-hydroxymellein	*D. eschscholtzii* HJ004 (the stem of *B. sexangula var. rhynchopetala*, endophytic fungus)	[103]
407	(*R*)-(-)-5-Carbonylmellein	*X. cubensis* PSU-MA34 (the branch of *B. parviflora*, endophytic fungus)	[97]
408	(*R*)-(-)-5-Methoxycarbonylmellein	*X. cubensis* PSU-MA34 (the branch of *B. parviflora*, endophytic fungus)	[97]
409	(*R*)-(-)-Mellein methyl ether	*X. cubensis* PSU-MA34 (the branch of *B. parviflora*, endophytic fungus)	[97]
410	Brugunin A	*B. gymnorrhiza*, branch	[126]
411	Brugnanin	*B. gymnorrhiza*, stem bark	[127]
412	Balanophonin	*B. gymnorrhiza*, hypocotyl	[124]
413	Secoisolariciresinol	*B. gymnorrhiza*, hypocotyl	[124]
414	Cleomiscosin A	*B. gymnorrhiza*, hypocotyl	[124]
415	Pinoresinol	*B. gymnorrhiza*, hypocotyl	[124]
416	Medioresinol	*B. gymnorrhiza*, hypocotyl	[124]
417	Lyoniresinol-3*α*-O-*β*-D-glucopyranosides	*B. gymnorrhiza*, hypocotyl	[124]
418	Aryl-tetralin lignan rhamnoside	*B. gymnorrhiza*, leaf	[119]
419	Rhyncosides E	*B. sexangula var. rhynchopetala*, stem	[128]
420	Rhyncosides F	*B. sexangula var. rhynchopetala*, stem	[128]

**Table 12:** Aromatic compounds isolated from *Bruguiera* genus plants and their endophytes.

No.	Compound	Source	Reference
421	Bruguierol A	*B. gymnorrhiza*, stem	[130]
422	Bruguierol B	*B. gymnorrhiza*, stem	[130]
423	Bruguierol C	*B. gymnorrhiza*, stem	[130]
424	1-(3-hydroxyphenyl)-hexane-2,5-diol	*B. gymnorrhiza*, stem	[130]
425	Bruguierol D	*B. gymnorrhiza*, branch	[126]
426	2,3-dimethoxy-5-propylphenol	*B. gymnorrhiza*, branch	[126]
427	Phenol A	*Penicillium sp.* 091402 (the root of *B. sexangula*, endophytic fungus)	[125]
428	3,4,5-trimethyl-1,2-benzenediol	*Penicillium sp.* 091402 (the root of *B. sexangula*, endophytic fungus)	[125]
429	Tyrosol	*D. eschscholtzii* PSU-STD57 (the leaf of *B. gymnorrhiza*, endophytic fungus)	[99]
430	3-hydroxy-4-methoxybenzoic acid	*B. gymnorrhiza*, hypocotyl	[131]
431	4-hydroxybenzoic acid	*B. gymnorrhiza*, hypocotyl	[131]
432	4-methoxybenylacetic acid	*B. gymnorrhiza*, hypocotyl	[131]
433	Di-(2-entylhexyl) phthalate	*B. gymnorrhiza*, hypocotyl	[131]
434	Dibutylphthalate	*B. gymnorrhiza*, hypocotyl; *P. thomi* (the root of *B. gymnorrhiza*, endophytic fungus)	[66,131]
435	Methyl caffeate	*B. gymnorrhiza*, hypocotyl	[131]
436	Methyl 3,5-dihydroxybenzoate	*B. gymnorrhiza*, hypocotyl	[131]
437	(*S*)-3-(3′,5′-dihydroxy-2′,4′-methylphenyl) butan-2-one	*Penicillium sp.* 091402 (the root of *B. sexangula*, endophytic fungus)	[125]
438	1-(2,6-dihydroxyphenyl)butan-1-one	*D. eschscholtzii* PSU-STD57 (the leaf of *B. gymnorrhiza*, endophytic fungus); *P. citrinum* HL-5126 (the leaf of *B. sexangula var. rhynchopetala*, endophytic fungus); *D. eschscholtzii* HJ001 (*B. sexangular var. rhynchopetala*, endophytic fungus)	[99,100,102]
439	1-(2-methoxyphenyl)butan-1-one	*P. longicolla* HL-2232 (the leaf of *B. sexangula var. rhynchopetala*, endophytic fungus)	[107]
440	Deoxyphomalone	*E. nigrum* MLY-3 (the leaf of *B. gymnorrhiza*, endophytic fungus)	[113]
441	Phomalone	*E. nigrum* MLY-3 (the leaf of *B. gymnorrhiza*, endophytic fungus)	[113]
442	Procyanidin	*B. parviflora*, bark	[132]
443	3-(3,4-dihydroxyphenyl)-7,8-dihydroxyhexahydro-6*H*-pyrano[2,3-b][1,4]dioxine-6-carboxylic acid	*B. gymnorrhiza*, leaf	[34]
444	2-(((3,4-dihydroxy-6-methyltetrahydro-2*H*-pyran-2-yl)oxy)methyl)-6-(3,4-dihydroxybenzyl)tetrahydro-2*H*-pyran-3,4,5-triol	*B. gymnorrhiza*, leaf	[34]
445	7,8-dihydroxy-3-(4-hydroxy-3-methoxyphenyl)-2-(hydroxymethyl)hexahydro-6*H*-pyrano[2,3-b][1,4]dioxine-6-carboxylic acid	*B. gymnorrhiza*, leaf	[34]
446	3-(3,4-dimethoxyphenyl)-7,8-dihydroxy-2-(hydroxymethyl)hexahydro-6*H*-pyrano[2,3-b][1,4]dioxine-6-carboxylic acid	*B. gymnorrhiza*, leaf	[34]
447	Rhyncosides A	*B. sexangula var. rhynchopetala*, stem	[128]
448	Rhyncosides B	*B. sexangula var. rhynchopetala*, stem	[128]
449	Rhyncosides C	*B. sexangula var. rhynchopetala*, stem	[128]
450	Rhyncosides D	*B. sexangula var. rhynchopetala*, stem	[128]
451	4′,5-dihydroxy-2,3-dimethoxy-4-(hydroxypropyl)-biphenyl	*P. thomi* (the root of *B. gymnorrhiza*, endophytic fungus)	[66]
452	5,5′-dimethoxybiphenyl-2,2′-diol	*P. longicolla* HL-2232 (the leaf of *B. sexangula var. rhynchopetala*, endophytic fungus)	[107]
453	6,6′-dimethoxybiphenyl-2,2′-diol	*P. longicolla* HL-2232 (the leaf of *B. sexangula var. rhynchopetala*, endophytic fungus)	[107]
454	3-(3-hydro-xybutyl)-1,1-dimethylisochroman-6,8-diol	*B. gymnorrhiza*, stem	[130]
455	(3*R*,4*S*)-6,8-dihydroxy-3,4,5,7-tetramethylisochroman	*Penicillium sp.* 091402 (the root of *B. sexangula*, endophytic fungus)	[125]
456	(1*S*,3*R*,4*S*)-1-(4′-hydroxylphenyl)-3,4-dihydro-3,4,5-trimethyl-1*H*-2-benzopyran-6,8-diol	*P. citrinum* (*B. gymnorrhiza*, endophytic fungus)	[117]
457	(2R*,4R*)-3,4-dihydro-5-methoxy-2-methyl-2*H*-1-benzopyran-4-ol	*P. citrinum* HL-5126 (the leaf of *B. sexangula var. rhynchopetala*, endophytic fungus)	[102]
458	(2R*,4R*)-3,4-dihydro-4-methoxy-2-methyl-2*H*-1-benzopyran-4-ol	*P. citrinum* HL-5126 (the leaf of *B. sexangula var. rhynchopetala*, endophytic fungus)	[102]
459	2-(2’*S*-hydroxypropyl)-5-methyl-7-hydroxychromone	*P. longicolla* HL-2232 (the leaf *B. sexangula var. rhynchopetala*, endophytic fungus)	[67]
460	5,7-dihydroxy-2-propylchromone	*P. citrinum* HL-5126 (the leaf of *B. sexangula var. rhynchopetala*, endophytic fungus)	[102]
461	Rhytidchromone A	*R. rufulum* (the leaf of *B. gymnorrhiza*, endophytic fungus)	[133]
462	Rhytidchromone B	*R. rufulum* (the leaf of *B. gymnorrhiza*, endophytic fungus)	[133]
463	Rhytidchromone C	*R. rufulum* (the leaf of *B. gymnorrhiza*, endophytic fungus)	[133]
464	Rhytidchromone D	*R. rufulum* (the leaf of *B. gymnorrhiza*, endophytic fungus)	[133]
465	Rhytidchromone E	*R. rufulum* (the leaf of *B. gymnorrhiza*, endophytic fungus)	[133]
466	Alternariol 5-O-methyl ether	*P. longicolla* HL-2232 (the leaf of *B. sexangula var. rhynchopetala*, endophytic fungus)	[107]
467	2-acetyl-7-methoxybenzofuran	*D. eschscholtzii* HJ004 (the stem of *B. sexangula var. rhynchopetala*, endophytic fungus)	[103]
468	4,6-dihydroxy-5-methoxy-7-methyl-1,3-dihydroisobenzofuran	*E. nigrum* MLY-3 (the leaf of *B. gymnorrhiza*, endophytic fungus)	[113]
469	Furobenzotropolone A	*E. nigrum* MLY-3 (the leaf of *B. gymnorrhiza*, endophytic fungus)	[113]
470	Furobenzotropolone B	*E. nigrum* MLY-3 (the leaf of *B. gymnorrhiza*, endophytic fungus)	[113]
471	3-hydroxyepicoccone B	*E. nigrum* MLY-3 (the leaf of *B. gymnorrhiza*, endophytic fungus)	[113]
472	4,6-dihydroxy5-methoxy-7-methylphthalide	*E. nigrum* MLY-3 (the leaf of *B. gymnorrhiza*, endophytic fungus)	[113]
473	4,5,6-trihydroxy-7-methyl-3*H*-isobenzofuran-1-one	*E. nigrum* MLY-3 (the leaf of *B. gymnorrhiza*, endophytic fungus)	[113]
474	Sparalide C	*E. nigrum* MLY-3 (the leaf of *B. gymnorrhiza*, endophytic fungus)	[113]

**Table 13:** Other compounds isolated from *Bruguiera* genus plants and their endophytes.

No.	Compound	Source	Reference
475	(9*Z*,12*Z*,15*Z*)-6,8,11-trihydroxyoctadeca-9,12,15-trienoic acid	*B. gymnorrhiza*, leaf	[34]
476	(9*E*,12*Z*)-6,8,11-trihydroxyoctadeca-9,12-dienoic acid	*B. gymnorrhiza*, leaf	[34]
477	(*E*)-6,8,12-trihydroxyoctadec-9-enoic acid	*B. gymnorrhiza*, leaf	[34]
478	8,12-dihydroxyhexadecanoic acid	*B. gymnorrhiza*, leaf	[34]
479	Tetradeca-5,8-dienoic acid	*S. albidoflavus* 107A-01824 (the leaf of *B. gymnorrhiza*, endophytic actinomycete)	[136]
480	Clonostach acids A	*C. rosea* B5-2 (the branch of *B. gymnorrhiza*, endophytic fungus)	[108]
481	Clonostach acids B	*C. rosea* B5-2 (the branch of *B. gymnorrhiza*, endophytic fungus)	[108]
482	Clonostach acids C	*C. rosea* B5-2 (the branch of *B. gymnorrhiza*, endophytic fungus)	[108]
483	Succinic acid	*P. thomi* (the root of *B. gymnorrhiza*, endophytic fungus)	[66]
484	Xylacinic acid A	*X. cubensis* PSU-MA34 (the branch of *B. parviflora*, endophytic fungus)	[97]
485	Xylacinic acid B	*X. cubensis* PSU-MA34 (the branch of *B. parviflora*, endophytic fungus)	[97]
486	2-Hexylidene-3-methyl succinic acid 4-methyl ester	*X. cubensis* PSU-MA34 (the branch of *B. parviflora*, endophytic fungus)	[97]
487	Prolipyrone A	*Fusarium proliferatum* MA-84 (the inner tissue of *B. sexangula*, endophytic fungus)	[45]
488	Prolipyrone B	*F. proliferatum* MA-84 (the inner tissue of *B. sexangula*, endophytic fungus)	[45]
489	Prolipyrone C	*F. proliferatum* MA-84 (the inner tissue of *B. sexangula*, endophytic fungus)	[45]
490	Gibepyrone D	*F. proliferatum* MA-84 (the inner tissue of *B. sexangula*, endophytic fungus)	[45]
491	Erythritol	*P. citrinum* ZD6 (the stem of *B. gymnorrhiza*, endophytic fungus)	[92]
492	Mannitol	*P. citrinum* ZD6 (the stem of *B. gymnorrhiza*, endophytic fungus)	[92]
493	Eicosanol	*B. cylindrica*, leaf	[58]
494	(2*R*,3*R*,4*S*,5*R*,6*R*)-4-(((4*R*,5*S*,6*R*)-4-hydroxy-5-methoxy-6-methyltetrahydro-2H-pyran-3-yl)oxy)-6-(hydroxymethyl)tetrahydro-2*H*-pyran-2,3,5-triol	*B. gymnorrhiza*, leaf	[34]
495	Cowabenzophenone A	*A. terreus* (*B. gymnorrhiza*, endophytic fungus)	[135]
496	(±)-1-monopalmitin	*P. thomi* (the root of *B. gymnorrhiza*, endophytic fungus)	[66]

**Table 14:** Cytotoxic activity of the compounds isolated from *Bruguiera* genus and its endophytes.

Compound	Cell Lines/Brine Shrimp	Effects	References
Acaciicolide B (**10**)	cytotoxic activity against MOLT-3 and HL-60 cancer cells;	IC_50_ values of 165.04 and 159.05 μM	[29]
3-epi-Steperoxide A (**27**)	cytotoxic activity against MOLT-3, HuCCA-1, A549, HepG2, HL-60, MDA-MB-231, T47D, and HeLa cancer cell lines;	IC_50_ values range of 0.68–3.71 μg/mL	[28]
Steperoxide A (**30**)	cytotoxic activity against MOLT-3, HuCCA-1, A549, HepG2, HL-60, MDA-MB-231, T47D, and HeLa cancer cell lines;	IC_50_ values range of 0.67–5.25 μg/mL	[28]
Merulin A (**31**)	cytotoxic activity against HuCCA-1, A549, MOLT-3, HepG2, HL-60, MDA-MB-231, T47D, Hela and MRC-5 cell lines;	IC_50_ values range of 15.20–76.97 μM	[29]
Merulin B (**32**)	cytotoxic activity against MOLT-3, A549, HepG2, HL-60, MDA-MB-231, and T47D cell lines;	IC_50_ values range of 11.94–49.08 μg/mL	[28]
Merulin C (**33**)	cytotoxic activity against HL-60 cancer cells; cytotoxic activity against MOLT-3, HuCCA-1, A549, HepG2, MDA-MB-231, T47D, and HeLa cell lines;	IC_50_ value of 0.08 μg/mL; IC_50_ values range of 0.19–3.75 μg/mL	[28]
Merulin D (**34**)	cytotoxic activity against HuCCA-1, A549, MOLT-3, HepG2, HL-60, MDA-MB-231, T47D, Hela and MRC-5 cell lines;	IC_50_ values range of 18.31–154.51 μM	[29]
7-epi-merulin B (**35**)	cytotoxic activity against HuCCA-1, A549, MOLT-3, HepG2, HL-60, MDA-MB-231, T47D, and MRC-5 cell lines;	IC_50_ values range of 0.28–37.46 μM	[29]
Botryosphaerin F (**40**)	cytotoxic activity against MCF-7 and HL-60 cancer cells;	IC_50_ values of 4.49 and 3.43 μM	[36]
13,14,15,16-tetranorlabd-7-ene-19,6b:12,17-diolide (**41**)	cytotoxic activity against MCF-7 and HL-60 cancer cells;	IC_50_ values of 2.79 and >30 μM	[36]
Botryosphaerin B (**42**)	cytotoxic activity against MCF-7 and HL-60 cancer cells;	IC_50_ values of 17.60 and >30 μM	[36]
LLZ1271*β* (**43**)	cytotoxic activity against MCF-7 and HL-60 cancer cells;	IC_50_ values of >30 and 0.60 μM	[36]
*Ent*-kaur-16-en-13-hydroxy-19-al (**64**)	cytotoxic activities against L-929, K562 and HeLa cell lines;	GI_50_/CC_50_ values of 11.5, 10.5 and 42.3 μg/mL	[42]
*Ent*-kaur-16-en-13,19-diol (**65**)	cytotoxic activities against L-929, K562 and HeLa cell lines;	GI_50_/CC_50_ values of 50.0, 50.0 and 50.0 μg/mL	[42]
17-chloro-13,16*β*-dihydroxy-*ent*-kauran-19-al (**68**)	cytotoxic activities against L-929, K562 and HeLa cell lines;	GI_50_/CC_50_ values of 50.0, 29.2 and 38.2 μg/mL	[42]
Methyl-16*α*,17-dihydroxy-*ent*-kauran-19-oate (**69**)	cytotoxic activities against L-929, K562 and HeLa cell lines;	GI_50_/CC_50_ values of 39.5, 27.7 and 40.5 μg/mL	[42]
Methyl-16*α*,17-dihydroxy-*ent*-9(11)-kauren-19-oate (**71**)	cytotoxic activities against L-929, K562 and HeLa cell lines;	GI_50_/CC_50_ values of 41.9, 26.7 and 38.7 μg/mL	[42]
16*α*,17-dihydroxy-*ent*-kauran-19-al (**72**)	cytotoxic activities against L-929, K562 and HeLa cell lines;	GI_50_/CC_50_ values of 50.0, 50.0 and 50.0 μg/mL	[42]
16*αH*-17-hydroxy-*ent*-kauran-19-oic acid (**73**)	cytotoxic activities against L-929, K562 and HeLa cell lines;	GI_50_/CC_50_ values of 42.4, 32.8 and 43.0 μg/mL	[42]
16*αH*-17,19-*ent*-kaurane-diol (**74**)	cytotoxic activities against L-929, K562 and HeLa cell lines;	GI_50_/CC_50_ values of 12.8, 13.6 and 35.7 μg/mL	[42]
16-*ent*-kauren-19-ol (**75**)	cytotoxic activities against L-929, K562 and HeLa cell lines;	GI_50_/CC_50_ values of 18.2, 6.8 and 32.8 μg/mL	[42]
(5*R*,9*S*,10*R*,13*S*,15*S*)-*ent*-8(14)-pimarene-1-oxo-15*R*,16-diol (**82**)	cytotoxic activities against L-929 cell lines;	IC_50_ value of 9.8 μg/mL	[43]
15(*S*)-isopimar-7-en-15,16-diol (**83**)	cytotoxic activities against K562 cell lines;	IC_50_ value of 7.0 μg/mL	[43]
(4*R*,5*S*,8*R*,9*R*,10*S*,13*S*)-*ent*-17-hydroxy-16-oxobeyeran-19-al (**85**)	cytotoxic activities against L-929, K562 and HeLa cell lines;	GI_50_/CC_50_ values of 45.4, 50.0 and 37.7 μg/mL	[42]
Sexangulic acid (**94**)	anti-tumour activity against A-549 and HL60 cell lines;	moderate in vitro cytotoxicity at 5 μg/mL	[47]
3*α*-*Z*-feruloyltaraxerol (**120**)	cytotoxic activities against NCI-H187 cell lines;	IC_50_ value of 12.2 μg/mL	[57]
3*α*-*Z*-coumaroyltaraxerol (**124**)	cytotoxic activities against NCI-H187 cell lines;	IC_50_ value of 20.0 μg/mL	[57]
3*β*-(*Z*)-coumaroyltaraxerol (**129**)	cytotoxic against Neuro 2A cell lines	IC_50_ value of 75.76 μM	[58]
Nectrianolin D (**144**)	cytotoxic against HL-60 cell lines;	IC_50_ value of 10.16 μM	[63]
Furanocochlioquinol (**145**)	cytotoxic against HL-60 cell lines;	IC_50_ value of 0.47 μM	[63]
Furanocochlioquinone (**146**)	cytotoxic against HL-60 cell lines;	IC_50_ value of 0.63 μM	[63]
Nectripenoid B (**147**)	cytotoxic against HL-60 cell lines;	IC_50_ value of 0.93 μM	[63]
Cochlioquinone D (**148**)	cytotoxic against HL-60 cell lines;	IC_50_ value of 1.61 μM	[63]
3*β*,5*α*-dihydroxy-(22*E*,24*R*)-ergosta-7,22-dien-6-one (**161**)	cytotoxic against MCF-7, A549, Hela and KB cell lines;	IC_50_ values of 4.98, 1.95, 0.68 and 1.50 μM	[69]
3*β*,5*α*,14*α*-trihydroxy-(22*E*,24*R*)-ergosta-7,22-dien-6-one (**162**)	cytotoxic against MCF-7, A549, Hela and KB cell lines;	IC_50_ values of 25.4, 27.1, 24.4 and 19.4 μM	[69]
Meleagrin (**193**)	cytotoxic against MCF-7, A549, Hela and KB cell lines;	IC_50_ values of 9.7 and 8.3 μM	[88]
Uridine (**194**)	cytotoxic against B16F10, A549, HL60 and MCF-7 cell lines;	IC_50_ values of 16.7, 8.6, 15.9 and 31.9 μmol/L	[67]
6-aminopurine-9-carboxylic acid methyl ester (**195**)	cytotoxic against B16F10, A549, HL60 and MCF-7 cell lines;	IC_50_ values of 29.0, 21.2, 4.1 and 14.9 μmol/L	[67]
Adenine riboside (**196**)	cytotoxic against B16F10, A549, HL60 and MCF-7 cell lines;	IC_50_ values of 18.2, 12.5, 14.4 and 26.2 μmol/L	[67]
Cyclo-(Ala-Gly) (**220**)	cytotoxic against K562, HepG2 and HT29 cell lines;	IC_50_ values of 18.1, 9.5, and 10.3 μM	[66]
Cyclo-(Pro-Gly) (**221**)	cytotoxic against K562, HepG2 and HT29 cell lines;	IC_50_ values of 17.6, >50 and 10.8 μM	[66]
Cyclo-(Ala-Pro) (**222**)	cytotoxic against K562, HepG2 and HT29 cell lines;	IC_50_ values of 9.6, 13.6, and 20.1 μM	[66]
Beauvericin (**226**)	cytotoxic against HL60 and A549 cell lines;	IC_50_ values of 2.02, 0.8, 1.14 and 1.10 μM	[69]
Palmarumycins BG5 (**236**)	cytotoxic against MCF-7 and HL-60 cell lines;	IC_50_ values of 7.6 and 1.9 μM	[98]
Nigronatthaphenyl (**242**)	cytotoxic against HCT 116 colon cell line;	IC_50_ value of 9.62 μM	[101]
Incarxanthone B (**258**)	cytotoxicity against A375, MCF-7 and HL-60 cell lines;	IC_50_ values of 8.6, 6.5, and 4.9 μM	[104]
Cytochalasin D (**296**)	cytotoxic against KB cell lines;	IC_50_ value of 3.99 μg/mL	[97]
Zygosporin D (**297**)	cytotoxic against HCT15 cell lines;	IC_50_ value of 13.5 μM	[110]
12-hydroxylcytochalasin Q (**305**)	cytotoxic against HCT15 cell lines;	IC_50_ value of 13.4 μM	[110]
Penibenzophenone B (**321**)	cytotoxic against A549 cell lines;	IC_50_ value of 15.7 μg/mL	[87]
Asterric acid (**324**)	cytotoxic against Hela cell lines;	IC_50_ value of 21.6 μg/mL	[87]
Scopoletin (**384**)	cytotoxic against Hela, A435, A549 and K562 cell lines;	IC_50_ values of 764.7, 593.4, 290.2 and 487.7 μg/mL	[124]
(3R*,4S*)-6,8-dihydroxy-3,4,7-trimethylisocoumarin (**387**)	cytotoxic against K562 cell lines;	IC_50_ value of 18.9 μg/mL	[125]
Brugnanin (**411**)	cytotoxic against CNE-1 nasopharyngeal carcinoma cell lines;	IC_50_ value of 5.72 × 10^−4^ M	[127]
Secoisolariciresinol (**413**)	cytotoxic against Hela, A435, A549 and K562 cell lines;	IC_50_ values of 571.5, 397.5, 323.0 and 768.8 μg/mL	[124]
Lyoniresinol-3*α*-O-*β*-D-glucopyranosides (**417**)	cytotoxic against Hela, A435, A549 and K562 cell lines;	IC_50_ values of 328.8, 455.5, 209.3 and 361.9 μg/mL	[124]
3,4,5-trimethyl-1,2-benzenediol (**428**)	cytotoxic against SGC-7901 cell lines;	IC_50_ value of 36.0 μg/mL	[125]
Dibutylphthalate (**434**)	cytotoxic against K562, HepG2 and HT29 cell lines;	IC_50_ values of 17.3, 15.2, and 11.1 μM	[66]
4′,5-dihydroxy-2,3-dimethoxy-4-(hydroxypropyl)-biphenyl (**451**)	cytotoxic against K562, HepG2 and HT29 cell lines;	IC_50_ values of 10.1, 12.2 and 8.9 μM	[66]
Rhytidchromone A (**461**)	cytotoxic against MCF-7 and Kato-3 cell lines;	IC_50_ values of 19.3 and 23.3 μM	[133]
Rhytidchromone B (**462**)	cytotoxic against Kato-3 cell lines;	IC_50_ value of 21.4 μM	[133]
Rhytidchromone D (**464**)	cytotoxic against Kato-3 cell lines;	IC_50_ value of 16.8 μM	[133]
Rhytidchromone E (**465**)	cytotoxic against MCF-7 and Kato-3 cell lines;	IC_50_ values of 17.7 and 16.0 μM	[133]
Cowabenzophenone A (**495**)	cytotoxic against HCT 116 colon cell line;	IC_50_ value of 10.1 μM	[135]
Fusaprolifin A (**87**)	The lethality activity against brine shrimp;	lethality rate of 49.5%, at 100 mg/mL	[45]
Fusaprolifin B (**88**)	The lethality activity against brine shrimp;	lethality rate of 9.6%, at 100 mg/mL	[45]
Macrosporin-2-O-(6′-acetyl)-α-D-glucopyranoside (**269**)	The lethality activity against brine shrimp;	LD_50_ value of 10 μM	[106]

**Table 15:** Antimicrobial activity of the compounds isolated from *Bruguiera* genus and its endophytes.

Compound	Pathogen	Effect	Reference
(3*R*,4*R*,6*R*,7*S*)-7-hydroxyl-3,7-dimethyl-oxabicyclo[3.3.1]nonan-2-one (**1**)	*B. cinerea*, and *P. nicotianae*	3.1 and 6.3 μg/mL	[26]
(3*R*,4*R*)-3-(7-methylcyclohexenyl)-propanoic acid (**2**)	*C. albicans*, *B. cinerea*, and *P. nicotianae*	50, 3.1 and 6.3 μg/mL	[26]
7*R*-hydroxygeosmin (**49**)	*B. subtilis*, *E. coli* and *P. aeruginosa*	weak activities	[32]
3-oxogeosmin (**50**)	*B. subtilis*, *P. aeruginosa* and VRE	weak activities	[32]
(4*S*,5*S*,7*R*,10*S*)-4*β*,10*α*-eudesmane-5*β*,11-diol (**53**)	*B. subtilis*, *S. aureus*, *E. coli*, *P. aeruginosa*, MRSA, VRE, *M. vaccae*, *S. salmonicolor* and *P. notatum*	broad antimicrobial activities	[32]
11-oxo-12*α*-acetoxy-4,4-dimethyl-24-methylene-5*α*-cholesta-8,14-diene-2*α*,3*β*-diol (**95**)	*S. aureus*, *B. subtilis*, *E. coli*, *M. luteus*, *M. tetragenus*, *S. albus* and *B. cereus*	>20, >20, 9.76, >20, >20, 9.76 and 9.76 μmol/L	[48]
12*α*-acetoxy-4,4-dimethyl-24-methylene-5*α*-cholesta-8-momoene-3*β*,11*β*-diol (**96**)	*S. aureus*, *B. subtilis*, *E. coli*, *M. luteus*, *M. tetragenus*, *S. albus* and *B. cereus*	5, 10, 5, >20, 5, 10 and 10 μmol/L	[48]
12*α*-acetoxy-4,4-dimethyl-24-methylene-5*α*-cholesta-8,14-diene-2*α*,3*β*,11*β*-triol (**98**)	*S. aureus*, *B. subtilis*, *E. coli*, *M. luteus*, *M. tetragenus*, *S. albus* and *B. cereus*	4.86, 9.72, 4.86, 9.72, 4.86, >20 and >20 μmol/L	[48]
Dehydroaustin (**131**)	*S. epidermidis*, *S. aureus*, *E. coli*, *B. cereus* and *V. alginolyticus*	20, >20, >20, >20 and >20 µM	[60]
11*β*-acetoxyisoaustinone (**132**)	*S. epidermidis*, *S. aureus*, *E. coli*, *B. cereus* and *V. alginolyticus*	20, >20, >20, >20 and >20 µM	[60]
Austinol (**133**)	*S. epidermidis*, *S. aureus*, *E. coli*, *B. cereus* and *V. alginolyticus*	10, >20, >20, 20 and >20 µM	[60]
Guignardone A (**149**)	*P. aeruginosa*, and MRSA	25, and 25 μg/mL	[64]
Guignardone J (**151**)	*P. aeruginosa*, and MRSA	50, and 50 μg/mL	[64]
BG138 (a mixture of brugierol **172** and isobrugierol **173**)	*P. aeruginosa*	32 μg/mL	[74]
Thiocladospolide A (**179**)	*E. tarda*, *E. ictarda* and *C. glecosporioides*	1, 8 and 2 µg/mL	[21]
Thiocladospolide B (**180**)	*C. glecosporioides*, *P. piricola* Nose and *F. oxysporum* f. sp. *Cucumerinum*	2, 32 and 1 µg/mL	[21]
Thiocladospolide C (**181**)	*C. glecosporioides*, *P. piricola* Nose and *F. oxysporum* f. sp. *Cucumerinum*	1, 32 and 32 µg/mL	[21]
Thiocladospolide D (**182**)	*E. ictarda*, *C. glecosporioides*, *P. piricola* Nose and *F. oxysporum* f. sp. *Cucumerinum*	1, 1, 32 and 1 µg/mL	[21]
Pandangolide 3 (**183**)	*C. glecosporioides* and *B. sorokiniana*	2 and 8 µg/mL	[21]
Thiocladospolide F (**184**)	*E. coli*, *E. tarda*, *P. aeruginosa*, *V. anguillarum*, *F. oxysporum* f. sp. *Momodicae*, *P. digitatum* and *H. maydis*	16, 1.0, 4.0, 2.0, 32, 32 and 8.0 µg/mL	[75]
Thiocladospolide G (**185**)	*E. coli*, *E. tarda*, *V. anguillarum*, *F. oxysporum* f. sp. *Momodicae*, *P. digitatum* and *H. maydis*	16, 2.0, 2.0, 16, 16 and 4.0 µg/mL	[75]
Antimycin A18 (**188**)	plant pathogenic fungi: *C. lindemuthianum*, *B. cinerea*, *A. solani* and *M. grisea*	the minimum concentration values to show inhibition zone on plates were 0.01, 0.06, 0.03 and 0.20 μg/mL	[86]
Penibruguieramine A (**199**)	*S. aureus*	20 µg/mL	[88]
Cyclo-(Ala-Gly) (**220**)	*B. subtilis*	400 µg/mL	[92]
2-Chloro-5-methoxy-3-methylcyclohexa-2,5-diene-1,4-dione (**231**)	*SA* and *MRSA*	equal MIC values of 128 µg/mL	[97]
8-methoxy-1-naphthol (**240**)	*S. aureus*, MRSA and *M. gypseum*	200 µg/mL	[99]
1,8-dimethoxynaphthalene (**241**)	*S. aureus*, MRSA and *M. gypseum*	200 µg/mL	[99]
Nigronatthaphenyl (**242**)	*B. subtilis*, *B. subtilis* TISTR 088, *B. cereus TISTR 688, S. aureus*, *E. coli*, MRSA, *C. albicans*, *P. aeruginosa*, *C. gloeosporioides* and *A. niger*	4, 4, 2, 4, 2, 2, 2, 2, 4 and 8 μg/mL	[101]
(3*S*)-3,8-Dihydroxy-6,7-dimethyl-*α*-tetralone (**243**)	*S. aureus*, MRSA *and M. gypseum*	200 µg/mL	[99]
Isosclerone (**244**)	*S. aureus*, MRSA and *M. gypseum*	200 µg/mL	[99]
1,3,8-trimethoxynaphtho[9-c]furan (**247**)	*S. aureus*, MRSA and *B. cereus*	>25, >25 and 12.5 µg/mL	[103]
5-hydroxy-2-methoxy-6,7-dimethyl-1,4-naphthoquinone (**255**)	*B. cereus*	12.5 µg/mL	[103]
Chloroisosulochrin dehydrate (**266**)	MRSA, *S. aureus*, *E. coli*, *B. cereus*, *V. alginolyticus* and *V. parahaemolyticus*	with the same MIC value of 50 Μm	[105]
Emodin (**267**)	*B. subtilis* and *P. aeruginosa*	25 and 100 µg/mL	[92]
Auxarthrol C (**268**)	*E. coli*	9.8 µM	[106]
Macrosporin (**270**)	*M. tetragenus*, *E. coli*, *S. albus*, *S. aureus* and *B. subtilis*	7.8, 3.9, 3.9, 3.9 and 3.9 µM	[106]
2-O-acetylaltersolanol B (**272**)	*M. tetragenus*, *E. coli* and *S. aureus*	4.6, 4.6 and 9.2 µM	[106]
Altersolanol A (**273**)	*M. tetragenus*, *E. coli*, *S. aureus* and *B. subtilis*	8.2, 4.1, 2.07 and 4.1 µM	[106]
Altersolanol B (**274**)	*V. parahaemolyticus* and *V. anguillarum*; *M. tetragenus*, *S. aureus*, *K. rhizophila*, and *B. subtilis*	2.5 and 5 µg/mL; 7.8, 7.8, 7.8 and 7.8 µM	[106,107]
Altersolanol C (**275**)	*B. subtilis*	8.8 µM	[106]
Tetrahydroaltersolanol B (**279**)	*E. coli*	7.3 µM	[106]
Alterporriol U (**282**)	*B. cereus*	8.3 µM	[106]
Alterporriol V (**283**)	*B. cereus*	8.1 µM	[106]
Alterporriol B (**286**)	*B. cereus*	7.9 µM	[106]
Alterporriol D (**287**)	*E. coli*, *B. cereus* and *S. aureus*	7.5, 10.0 and 5.0 µM	[106]
Alterporriol E (**288**)	*E. coli* and *B. cereus*	5.0 and 2.50 µM	[106]
Alterporriol C (**289**)	*B. cereus*	8.9 µM	[106]
2′-acetoxy-7-chlorocitreorosein (**293**)	*S. aureus* and *V. parahaemolyticus*	22.8 and 10 µM	[105]
Citreorosein (**294**)	*S. aureus*, *E. coli*, *B. cereus*, *V. alginolyticus* and *V. parahaemolyticus*	22.8, 50, 50, 50 and 50 µM	[105]
MT-1 (**295**)	MRSA, *S. aureus*, *E. coli*, *B. cereus*, *V. alginolyticus* and *V. parahaemolyticus*	with the same MIC value of 50 Μm	[105]
[11]-cytochalasa-5(6),13-diene-1,21-dione-7,18-dihydroxy-16,18-dimethyl-10-phenyl(7S*,13*E*,16S*,18R*) (**298**)	*E. coli*, *S. aureus*, *B. cereus*, *V. parahaemolyticus* and *V. alginolyticus*	with the same MIC value of 50 μg/mL	[100]
2-deoxy-sohirnone C (**317**)	MRSA	80 μg/mL	[90]
3,4-dihydroxybenzoic acid (**319**)	*P. aeruginosa*, *E. faecalis*, MRSA, *E. coli* and *C. albicans*	25, 25, 25, 12.5 and 25 μg/mL	[64]
8-O-methylnodulisporin F (**326**)	*S. aureus*, MRSA and *B. cereus*	6.25, 12.5 and 6.25 µg/mL	[103]
Nodulisporin H (**327**)	*S. aureus*, MRSA and *B. cereus*	12.5, 12.5 and 6.25 µg/mL	[103]
5*R*-hydroxyrecifeiolide (**338**)	*E. ictarda* and *P. aeruginosa*	32 and 32 µg/mL	[114]
5*S*-hydroxyrecifeiolide (**339**)	*G. cingulate*	16 µg/mL	[114]
Pandangolide 1 (**340**)	*S. aureus*, *E. ictarda*, *G. cingulate* and *P. aeruginosa*	32, 4.0, 1.0 and 32 µg/mL	[114]
Cladocladosin A (**341**)	*E. tarda*, *V. anguillarum*, *F. oxysporum* f. sp. *Momodicae*, *P. digitatum* and *H. maydis*	2.0, 4.0, 32, 32 and 8.0 µg/mL	[75]
Infectopyrone A (**345**)	*S. albus*, *E. coli*, *B. subtilis*, *M. tetragenus* and *M. luteus*	5.0, 2.5, 10.0, 10.0 and 10.0 µg/mL	[106]
Infectopyrone B (**346**)	*S. albus*, *E. coli*, *M. tetragenus* and *M. luteus*	10.0, 2.5, 10.0 and 10.0 µg/mL	[106]
6,8-dihydroxy-5-methoxy-3-methyl-1H-isochromen-1-one (**347**)	*P. aeruginosa*, and MRSA	50, and 50 μg/mL	[64]
*Ent*-cladospolide F (**348**)	*S. aureus*, *E. ictarda* and *P. aeruginosa*	8.0, 16 and 64 µg/mL	[114]
Cladospolide G (**349**)	*E. coli*, *G. cingulate*, *B. sorokiniana* and *F. oxysporum* f. sp. *Cucumerinum*	32, 1.0, 32 and 1.0 µg/mL	[114]
Iso-cladospolide B (**351**)	*E. coli*, *E. tarda*, *E. ictarda* and *G. cingulate*	32, 32, 16 and 64 µg/mL	[114]
Penicimarin G (**390**)	*S. epidermidis*, *S. aureus*, *E. coli*, *B. cereus* and *V. alginolyticus*	20, 20, 20, 20 and 20 µM	[60]
Penicimarin H (**391**)	*S. epidermidis*, *S. aureus*, *E. coli*, *B. cereus* and *V. alginolyticus*	10, 20, >20, 20 and 20 µM	[60]
Bruguierol A (**421**)	*M. vaccae* and *C. albicans*	25 and 50 µg/mL	[130]
Bruguierol B (**422**)	*M. vaccae* and *C. albicans*	25 and 50 µg/mL	[130]
Bruguierol C (**423**)	*S. aureus*, *M. luteus*, *E. faecalis*, *E. coli*, *M. vaccae* and *C. albicans*	12.5, 12.5, 12.5, 12.5, 12.5 and 50 µg/mL	[130]
Tyrosol (**429**)	*S. aureus*, MRSA and *M. gypseum*	200 µg/mL	[99]
1-(2,6-dihydroxyphenyl)butan-1-one (**438**)	*S. aureu*s, MRSA and *M. gypseum;* *B. subtilis*, *B. cereus* and *M. tetragenus*	200 µg/mL; 6.94 µM	[99]
5,5′-dimethoxybiphenyl-2,2′-diol (**452**)	*V. parahaemolyticus*	10 µg/mL	[107]
2-(2′*S*-hydroxypropyl)-5-methyl-7-hydroxychromone (**459**)	*E. coli*	5 µg/mL	[67]
Erythritol (**491**)	*B. subtilis*	50 µg/mL	[58]
Cowabenzophenone A (**495**)	*B. subtilis*, *S. aureus*, *E. coli*, MRSA, *C. albicans*, *P. aeruginosa* and *C. gloeosporioides*	1, 2, 4, 4, 4, 2 and 2 μg/mL	[135]

Abbreviations: *A. niger* = *Aspergillus niger*; *A. solani* = *Alternaria solani*; *B. cereus* = *Bacillus cereus*; *B. subtilis* = *Bacillus subtilis*; *B. cinerea* = *Botrytis cinerea*; *C. gloeosporioides* = *Colletotrichum gloeosporioides*; *C. lindemuthianum* = *Colletotrichum lindemuthianum*; *E. faecalis* = *Enterococcus faecalis*; *E. coli* = *Escherichia coli*; *F. oxysporum* f. sp. *Momodicae* = *Fusarium oxysporum* f. sp. *Momodicae*; *G. cingulate* = *Glomerella cingulate*; *H. maydis* = *Helminthosporium maydis*; *K. rhizophila* = *Kocuria rhizophila*; MRSA = methicillin-resistant *Staphylococcus aureus*; *M. grisea* = *Magnaporth grisea*; *M. luteus* = *Micrococcus luteus*; *M. tetragenus* = *Micrococcus tetragenus*; *M. gypseum* = *Microsporum gypseum*; *M. vaccae* = *Mycobacterium vaccae*; *P. digitatum* = *Penicillium digitatum*; *P. notatum* = *Penicillium notatum*; *P. nicotianae* = *Phytophthora nicotianae*; *S. albus* = *Staphylococcus albus*; *S. epidermidis* = *Staphylococcus epidermidis*; *V. anguillarum* = *Vibrio anguillarum*; *S. salmonicolor* = *Sporobolomyces salmonicolor*; VRE = vancomycin-resistant *Enterococcus*; *V. alginolyticus* = *Vibrio alginolyticus*; *V. anguillarum* = *Vibrio anguillarum*; *V. parahaemolyticus* = *Vibrio parahaemolyticus*.

## Data Availability

No new data were created or analyzed in this study. Data sharing is not applicable to this article.
